# Microwave-assisted synthesis, computational studies and antibacterial/ anti-inflammatory activities of compounds based on coumarin-pyrazole hybrid

**DOI:** 10.1098/rsos.172435

**Published:** 2018-05-02

**Authors:** Rakesh R. Chavan, Kallappa M. Hosamani

**Affiliations:** Department of Studies in Chemistry, Karnataka University, Dharwad 580003, India

**Keywords:** microwave synthesis, Schiff base, coumarin, pyrazole, antibacterial, anti-inflammatory

## Abstract

An efficient, high-yield and rapid synthesis of (E)-1,5-dimethyl-4-((2-((substituted-2-oxo-2H-chromen-4-yl)methoxy)naphthalen-1-yl)methyleneamino)-2-phenyl-1,2-dihydropyrazol-3-one derivatives **(3a–3i)** containing Schiff base structures under microwave-irradiation has been described. Schiff base is a potential target to discover anti-inflammatory chemotherapeutics, material science, catalysis and molecular magnetism. All the newly synthesized compounds **(3a–3i)** have been characterized by elemental analysis and spectroscopic techniques. The synthesized compounds **(3a–3i)** were evaluated for their antibacterial activity by agar-well diffusion method and anti-inflammatory activity by egg albumin denaturation method. The compounds **(3e)** and **(3i)** exhibit antibacterial effect with minimum inhibitory concentration (MIC) 0.78 µg ml^−1^ and MIC 1.562 µg ml^−1^ against Gram-positive *Staphylococcus aureus* bacterial strain compared with standard ciprofloxacin drug (MIC 6.25 µg ml^−1^). The compounds **(3c)** and **(3f)** exhibited an inhibition of heat-induced protein denaturation at the concentration (31.25 µg ml^−1^) as 53.65% and 67.27%, respectively, and these compounds are more active than standard aceclofenac drug (5.50%). Molecular docking study has been performed for all the synthesized compounds with *S. aureus* dihydropteroate synthetase and results obtained are quite promising.

## 1. Introduction

Microbial infections are becoming the most pressing issue for global health and the economy [1]. In recent years, the treatment of bacterial infections has become a major challenge in the realm of conventional antibiotic therapy [2]. The emergence of bacterial resistance to established antibiotics, as well as hospital-acquired infections, causes a growing concern for the global community [3]. Thus, increasing resistance of microorganisms to currently available antimicrobial drugs is the major cause of morbidity and mortality throughout the world [4]. Microbial diseases, such as plague, diphtheria, typhoid, cholera, pneumonia and tuberculosis, have taken a high toll of humanity in the recent past [5]. Also, a number of recent clinical reports describe the increasing occurrence of methicillin-resistant *Staphylococcus aureus* (MRSA), *Staphylococcus epidermidis* and vancomycin-resistant enterococci, which are the leading causes of death from bacterial infections in most developed countries [6,7]. According to the World Health Organization (WHO), the infections caused by resistant microbes often fail to respond to conventional antibiotic therapy, resulting in prolonged illness and greater risk of death [8]. Thus, developments of novel antimicrobial drugs, which are distinct from those of well-known classes of antibacterial agents, are still in demand [9]. The microwave-assisted synthesis is very attractive for chemical applications and has become a widely accepted non-conventional energy source for the preparation of novel heterocyclic compounds [10].

**Figure 1.**
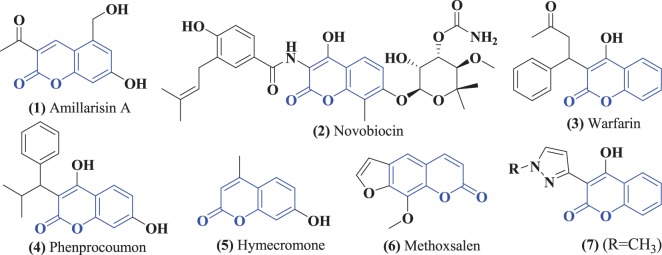
Structures of some coumarin derivatives which are commercially available in market.

Coumarin analogues are a group of bioactive molecules, found extensively in nature with a wide range of structural modifications. They belong to the family of lactones having benzopyrone system [11]. The biological activities of coumarin derivatives include antiviral, anti-cancer, antimicrobial, anti-inflammatory, anti-HIV, antioxidant, antituberculosis, anti-influenza and anticoagulant activities [12]. A few potent coumarin derivatives which are commercially available in market are Amillarisin A (antibiotic) **(1)**, Novobiocin (antibiotic) **(2)**, Warfarin (anticoagulant) **(3)**, Phenprocoumon (anticoagulant) **(4)**, Hymecromone (choleretic and antispasmodic) **(5)** and Methoxsalen (antipsoriasis) **(6)** (figure 1) [13]. Coumarin-pyrazole hybrids **(7)** have also potent *in vitro* antimicrobial activities against Gram-positive bacteria, *Staphylococcus aureus*, *Bacillus cereus* and Gram-negative *Salmonella*, at a concentration of 1 mg ml^−1^ [14].

**Figure 2.**
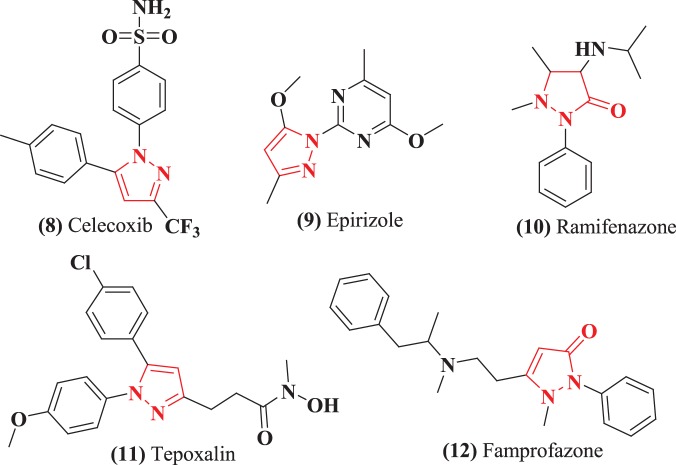
Chemical structures of anti-inflammatory agents (pyrazole derivative).

Pyrazole and its derivatives are important nitrogen-containing heterocyclic compounds. Pyrazole ring is a ubiquitous motif in biologically active compounds and therefore represents an interesting template for combinatorial medicinal chemistry [15] due to their capability to exhibit a wide range of bioactivities such as antimicrobial [16], anti-cancer [17], anti-inflammatory [18], antidepressant [19], anticonvulsant [20], antipyretic [21] and selective enzyme inhibitory activities [22]. Antipyrine was the first pyrazolone derivative used in the management of pain and inflammation, and their derivatives have attracted the attention of several research groups due to their potential activities [23]. The well-known anti-inflammatory drug, Celecoxib, is a pyrazole derivative; some examples of pyrazole derivative as non-steroidal anti-inflammatory drugs (NSAIDs) are Celecoxib **(8)**, Epirizole **(9)**, Ramifenazone **(10)**, Tepoxalin **(11)** and Famprofazone **(12)** (figure 2) [24].

Schiff base products are generally known as azomethine or imine compounds due to the presence of azomethine bond and are derived from the condensation of a primary amine with active carbonyl compound and generally take place under acid, base catalysis or with heat [25]. Schiff base is very widely used and the most appreciated organic building block to have a diverse range of pharmacological importance as well as a versatile tool to explore in biological, clinical, analytical and industrial applications etc. [26]. They hold a spectrum of biological importance as antioxidant, anthelmintic, antitubercular, anti-inflammatory, anti-cancer, antimicrobial and anticonvulsant [27]. The active centres of cell constituents are supposed to get interacted with azomethine's nitrogen atom via forming a hydrogen bond which interferes with normal cell processes [28] and results in the destruction of enzymatic activity of cancerous cells, thereby presents Schiff base as a potential target to discover anti-cancer chemotherapeutics. In recent years, the properties of Schiff base have generated much attention due to their properties that enhance works in material science, catalysis (carbonization, reduction and oxidation), molecular magnetism [29]. The use of Schiff bases as corrosion inhibitors has been studied [30].

It was also envisaged that coumarin and pyrazole pharmacophores, if linked together, would generate novel molecular templates which are likely to exhibit interesting biological properties in animal models. In particular, those pyrazole pharmacophores linked to coumarin have been reported to possess antimicrobial, analgesic, anti-inflammatory, antipyretic, anti-cancer and vascorelaxant properties [31].

Computational biology and bioinformatics play a major role in drug designing and accelerating the drug discovery process. Molecular docking of the drug molecule with the receptor (target) offers important information about drug-receptor interactions and is commonly employed to identify the binding orientation of drug molecules to their protein targets in order to predict the affinity and activity [32].

**Figure 3.**
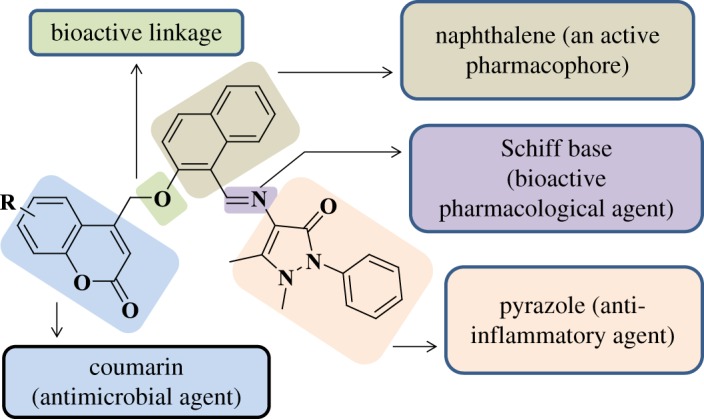
Designed hypothetical interaction module.

Our efforts focused on the introduction of chemical diversity in the molecular framework in order to synthesize pharmacologically interesting compounds of different composition. This motivated us to design and synthesize new coumarin templates bearing pyrazole moieties via ether linkage. Expectedly, the additive effect of this combination might produce a synergistic effect in enhancing the bioactivity of the coumarin derivatives. Thus, the designing of hypothetical interaction module is represented in figure 3. To further our continued effort towards the development of microwave-assisted synthetic methodologies [33], we describe in this paper an efficient and facile synthesis of coumarin-pyrazole hybrids **(3a–3i)** under microwave-irradiation, through which the yields of the compounds were improved drastically in a very short reaction time when compared with conventional methods.

## 2. Material and methods

### 2.1. Experimental

All reagents were of analytical grade and were used directly. Thin-layer chromatography (TLC) was performed on silica gel plates (60 F254; Merck). Melting points were determined using an open capillary method on a Buchi apparatus and are uncorrected. IR spectra were recorded on a Nicolet 6700 FT-IR instrument (Nicolet, Madison, WI, USA) using KBr pellets. ^1^H and ^13^C NMR spectra were recorded on Bruker 400** **MHz FT NMR spectrometer using CDCl_3_ and DMSO-d_6_ as solvent and TMS as internal standard. All chemical shifts were reported as δ values (ppm). Mass spectra were recorded using Shimadzu GCMSQP2010S. The elemental analysis was carried out using Hereaus CHN rapid analyser. Microwave irradiation synthesis was carried out under CEM-Discover Focused Microwave system. Purity of the compound was checked by TLC.

### 2.2. General procedure for the synthesis of (E)-4-((2-hydroxynaphthalen-1-yl)methyleneamino)-1,5-dimethyl-2-phenyl-1,2-dihydropyrazol-3-one **(1)**

A mixture of 2-hydroxy-1-naphthaldehyde (0.0025 mol), 4-amino-1,5-dimethyl-2-phenyl-1,2-dihydropyrazol-3-one (0.0025** **mol) and acetic acid (0.5 ml) was refluxed for 3 h in dry ethanol (30 ml). The mixture was cooled to room temperature and the obtained precipitate was filtered out and washed several times with cold ethanol and recrystallized from ethanol and dried over anhydrous calcium chloride under vacuum.

### 2.3. General procedure for the synthesis of compounds **(3a–3i)** by conventional method

A mixture of anhydrous potassium carbonate (0.00625 mol) and (E)-4-((2-hydroxynaphthalen-1-yl)methyleneamino)-1,5-dimethyl-2-phenyl-1,2-dihydropyrazol-3-one (**1**) (0.0025 mol) was stirred for 30 min in dry acetone (30 ml). To this, 4-bromomethyl coumarins (**2a–2i**) (0.0025 mol) were added and the stirring was continued for 4–6 h at room temperature. The mixture was diluted with crushed ice. Separated solid was filtered and washed with water, then with dilute HCl (1 : 1) and with water thoroughly. Solids were crystallized from DMF.

### 2.4. General procedure for the synthesis of compounds **(3a–3i)** under microwave-irradiation

A mixture of anhydrous potassium carbonate (0.0025 mol) and (E)-4-((2-hydroxynaphthalen-1-yl)methyleneamino)-1,5-dimethyl-2-phenyl-1,2-dihydropyrazol-3-one (**1**) (0.001 mol) was taken in dry acetone (3 ml). To this, 4-bromomethyl coumarins (**2a–2i**) (0.001 mol) were added into 10 ml microwave pressure vial and irradiated in a microwave reactor (Model: CEM-Discover Focused Microwave system) under 200 W power at 35°C for 8–12 min. The progress of reaction was monitored by TLC and the mixture was diluted with crushed ice. Separated solid was filtered and washed with water, then with dilute HCl (1 : 1) and with water thoroughly. Solids were crystallized from DMF.

#### 2.4.1. (E)-4-((2-((5,7-dimethyl-2-oxo-2H-chromen-4-yl)methoxy)naphthalen-1-yl)methylene amino)-1,5-dimethyl-2-phenyl-1,2-dihydropyrazol-3-one **(3a)**

Yellow solid; Mp 220–222°C; IR (KBr) (*v*_max_ cm^−1^): 1716 (C = O of lactone), 3424 (NH stretching); ^1^H NMR (400** **MHz, CDCl_3_, *δ* ppm) *δ* 2.37 (s, 3H, C_7_-CH_3_), 2.52 (s, 3H, CH_3_), 2.64 (s, 3H, C_5_-CH_3_), 3.18 (s, 3H, N-CH_3_), 5.52 (s, 2H, CH_2_), 6.86 (s, 1H, C_3_-H), 7.04 (s, 1H, Ar-H), 7.16 (d, 1H, *J* = 8.8 Hz, Ar-H), 7.28–7.32 (m, 1H, Ar-H), 7.39–7.50 (m, 5H, Ar-H), 7.52-7.56 (m, 1H, Ar-H), 7.78 (d, 1H, *J* = 8 Hz, Ar -H), 7.82 (d, 1H, *J* = 9.2 Hz, Ar -H), 9.22 (d, 1H, *J* = 8.4 Hz, Ar -H), 10.54 (s, 1H, -N = CH); ^13^C NMR (100 MHz, DMSO-d_6_, *δ* ppm): 14.56, 18.13, 21.58, 36.38, 59.66, 113.59, 115.43, 115.48, 115.81, 117.15, 117.39, 124.17, 124.99, 125.50, 125.95, 126.25, 127.04, 128.07, 128.77, 128.89, 134.26, 138.75, 139.24, 143.16, 143.59, 150.67, 152.31, 153.61, 154.09, 158.67, 159.99, 166.53; MS: 543[M]^+^; Anal. calcd for C_34_H_29_N_3_O_4_: C, 75.12; H, 5.38; N, 7.73%. Found: C, 75.03; H, 5.29; N, 7.64%.

#### 2.4.2. (E)-4-((2-((6-tert-butyl-2-oxo-2H-chromen-4-yl)methoxy)naphthalen-1-yl)methylene amino)-1,5-dimethyl-2-phenyl-1,2-dihydropyrazol-3-one **(3b)**

Yellow solid; Mp 218-220°C; IR (KBr) (*v*_max_ cm^−1^): 1724 (C = O of lactone), 3444 (NH stretching); ^1^H NMR (400** **MHz, CDCl_3_, *δ* ppm) *δ* 1.32 (s, 9H, ter-butyl), 2.51 (s, 3H, CH_3_), 3.18 (s, 3H, N-CH_3_), 5.48 (s, 2H, CH_2_), 6.83 (s, 1H, C_3_-H), 7.17–7.37 (m, 3H, Ar-H), 7.39–7.62 (m, 9H, Ar-H), 7.74–7.89 (m, 1H, Ar-H), 9.26 (d, 1H, *J* = 8.4** **Hz, Ar -H), 10.57 (s, 1H, -N = CH); ^13^C NMR (100** **MHz, CDCl_3_, *δ* ppm): 10.51, 31.45, 34.78, 36.06, 68.08, 113.44, 115.22, 116.58, 117.03, 119.25, 119.96, 120.80, 124.40, 124.73, 125.93, 126.86, 127.89, 128.35, 129.29, 129.69, 130.30, 132.30, 134.93, 135.33, 147.53, 150.54, 151.72, 152.13, 155.74, 156.34, 160.78, 160.92; MS: 571[M]^+^; Anal. calcd for C_36_H_33_N_3_O_4_: C, 75.64; H, 5.82; N, 7.35%. Found: C, 75.53; H, 5.75; N, 7.28%.

#### 2.4.3. (E)-4-((2-((6-methoxy-2-oxo-2H-chromen-4-yl)methoxy)naphthalen-1-yl)methylene amino)-1,5-dimethyl-2-phenyl-1,2-dihydropyrazol-3-one **(3c)**

Light yellow solid; Mp 198–200**°**C; IR (KBr) (*v*_max_ cm^−1^): 1720 (C = O of lactone), 3442 (NH stretching); ^1^H NMR (400** **MHz, DMSO-d_6_, *δ* ppm) *δ* 2.04 (s, 3H, CH_3_), 3.17 (s, 3H, N-CH_3_), 3.80 (s, 3H, C_6_-OCH_3_), 5.65 (s, 2H, CH_2_), 6.74 (s, 1H, C_3_-H), 7.21 (d, 1H, *J* = 9.2** **Hz, Ar-H), 7.31–7.46 (m, 6H, Ar-H), 7.48–7.62 (m, 3H, Ar-H), 7.74 (d, 1H, *J* = 9.2** **Hz, Ar-H), 7.90 (d, 1H, *J* = 8.4** **Hz, Ar-H), 8.04 (d, 1H, *J* = 9.2** **Hz, Ar -H), 9.41 (d, 1H, *J* = 8.8** **Hz, Ar-H), 10.45 (s, 1H, -N = CH); ^13^C NMR (100** **MHz, DMSO-d_6_, *δ* ppm): 10.61, 36.13, 56.42, 66.94, 108.19, 112.87, 115.41, 117.80, 118.19, 118.48, 118.61, 119.99, 124.80, 126.02, 127.17, 128.43, 128.96, 129.63, 129.76, 131.76, 132.94, 135.19, 142.33, 147.88, 151.41, 152.76, 154.02, 156.14, 156.80, 160.00, 160.24; MS: 545[M]^+^; Anal. calcd for C_33_H_27_N_3_O_5_: C, 72.65; H, 4.99; N, 7.70%. Found: C, 72.58; H, 4.87; N, 7.63%.

#### 2.4.4. (E)-1,5-dimethyl-4-((2-((6-methyl-2-oxo-2H-chromen-4-yl)methoxy)naphthalen-1-yl) methyleneamino)-2-phenyl-1,2-dihydropyrazol-3-one **(3d)**

Yellow solid; Mp 248-250**°**C; IR (KBr) (*v*_max_ cm^−1^): 1720 (C = O of lactone), 3443 (NH stretching); ^1^H NMR (400** **MHz, CDCl_3_, *δ* ppm) *δ* 2.16 (s, 3H, CH_3_), 2.45 (s, 3H, N-CH_3_), 4.46 (s, 2H, CH_2_), 6.44 (s, 1H, C_3_-H), 7.09–7.21 (m, 4H, Ar-H), 7.48–8.26 (m, 12H, Ar-H), 9.09–9.26 (m, 1H, Ar-H), 10.95 (s, 1H, -N = CH); ^13^C NMR (100** **MHz, CDCl_3_, *δ* ppm): 21.78, 26.86, 31.06, 67.46, 112.56, 114.86, 115.18, 116.48, 117.72, 118.58, 119.07, 124.25, 125.73, 128.73, 130.75, 132.24, 134.59, 138.00, 141.02, 143.81, 144.62, 144.90, 145.52, 146.52, 147.35, 148.50, 150.06, 154.21, 159.25, 160.67, 161.97; MS: 529[M]^+^; Anal. calcd for C_33_H_27_N_3_O_4_: C, 74.84; H, 5.14; N, 7.93%. Found: C, 74.78; H, 5.02; N, 7.71%.

#### 2.4.5. (E)-4-((2-((6-chloro-2-oxo-2H-chromen-4-yl)methoxy)naphthalen-1-yl)methylene amino)-1,5-dimethyl-2-phenyl-1,2-dihydropyrazol-3-one **(3e)**

Light brown solid; Mp 238-240**°**C; IR (KBr) (*v*_max_ cm^−1^): 1728 (C = O of lactone), 3446 (NH stretching); ^1^H NMR (400** **MHz, CDCl_3_, *δ* ppm) *δ* 2.46 (s, 3H, CH_3_), 3.20 (s, 3H, N-CH_3_), 5.71 (s, 2H, CH_2_), 6.98 (s, 1H, C_3_-H), 7.14 (d, 1H, *J* = 9.2 Hz, Ar-H), 7.28–7.52 (m, 7H, Ar-H), 7.71–7.86 (m, 5H, Ar-H), 8.22 (d, 1H, *J* = 8.4 Hz, Ar-H), 10.81 (s, 1H, -N = CH); ^13^C NMR (100 MHz, CDCl_3_, *δ* ppm): 14.27, 31.24, 69.68, 108.77, 112.93, 114.00, 114.94, 118.12, 121.31, 122.50, 126.22, 126.30, 129.08, 129.24, 129.48, 129.86, 130.10, 130.23, 131.46, 134.62, 134.75, 134.94, 138.59, 139.37, 141.43, 152.83, 154.89, 157.37, 160.18, 165.55; MS: 550[M]^+^; Anal. calcd for C_32_H_24_ClN_3_O_4_: C, 69.88; H, 4.40; N, 7.64%. Found: C, 69.65; H, 4.34; N, 7.58%.

#### 2.4.6. (E)-4-((2-((7-hydroxy-2-oxo-2H-chromen-4-yl)methoxy)naphthalen-1-yl)methylene amino)-1,5-dimethyl-2-phenyl-1,2-dihydropyrazol-3-one **(3f)**

Light brown solid; Mp 226-228°C; IR (KBr) (*v*_max_ cm^−1^): 1715 (C = O of lactone), 3444 (NH stretching); ^1^H NMR (400 MHz, DMSO-d_6_, *δ* ppm) *δ* 2.41 (s, 3H, CH_3_), 3.20 (s, 3H, N-CH_3_), 5.72 (s, 2H, CH_2_), 6.84 (s, 1H, C_3_-H), 7.14 (d, 1H, *J* = 8.4 Hz, Ar-H), 7.33–7.41 (m, 5H, Ar-H), 7.50–7.56 (m, 5H, Ar-H), 7.84 (d, 1H, *J* = 7.2 Hz, Ar-H), 7.90 (d, 1H, *J* = 9.2 Hz, Ar-H), 8.04 (d, 1H, *J* = 8.8 Hz, Ar -H), 10.62 (s, 1H, -N = CH), 14.93 (s, 1H, -OH); ^13^C NMR (100 MHz, DMSO-d_6_, *δ* ppm): 10.45, 35.78, 76.39, 107.64, 110.35, 110.42, 114.49, 119.79, 120.14, 120.38, 124.04, 125.53, 127.87, 128.05, 128.43, 129.65, 129.77, 130.07, 132.46, 134.02, 134.64, 136.94, 142.26, 143.72, 150.00, 154.63, 155.16, 159.80, 161.41, 162.08; MS: 531[M]^+^; Anal. calcd for C_32_H_25_N_3_O_5_: C, 72.30; H, 4.74; N, 7.91%. Found: C, 72.26; H, 4.61; N, 7.83%.

#### 2.4.7. (E)-1,5-dimethyl-4-((2-((7-methyl-2-oxo-2H-chromen-4-yl)methoxy)naphthalen-1-yl) methyleneamino)-2-phenyl-1,2-dihydropyrazol-3-one **(3g)**

Yellow solid; Mp 194–196°C; IR (KBr) (*v*_max_ cm^−1^): 1723 (C = O of lactone), 3426 (NH stretching); ^1^H NMR (400 MHz, CDCl_3_, *δ* ppm) *δ* 2.17 (s, 3H, CH_3_), 2.46 (s, 3H, N-CH_3_), 4.50 (s, 2H, CH_2_), 6.42 (s, 1H, C_3_-H), 7.11–7.21 (m, 4H, Ar-H), 7.36–8.22 (m, 12H, Ar-H), 9.09–9.26 (m, 1H, Ar-H), 10.95 (s, 1H, -N = CH); ^13^C NMR (100 MHz, CDCl_3_, *δ* ppm): 14.19, 21.23, 31.06, 59.91, 115.29, 115.41, 116.34, 123.29, 125.01, 125.31, 125.48, 125.59, 125.84, 127.91, 128.41, 129.97, 130.88, 131.20, 134.16, 135.65, 138.37, 138.57, 141.95, 145.58, 148.09, 149.95, 152.05, 155.25, 160.58, 161.28, 166.74; MS: 529[M]^+^; Anal. calcd for C_33_H_27_N_3_O_4_: C, 74.84; H, 5.14; N, 7.93%. Found: C, 74.78; H, 5.03; N, 7.87%.

#### 2.4.8. (E)-1,5-dimethyl-4-((2-((3-oxo-3H-benzo[f]chromen-1-yl)methoxy)naphthalen-1-yl) methyleneamino)-2-phenyl-1,2-dihydropyrazol-3-one **(3h)**

Yellow solid; Mp 236-238**°**C; IR (KBr) (*v*_max_ cm^−1^): 1731 (C = O of lactone), 3443 (NH stretching); ^1^H NMR (400 MHz, CDCl_3_, *δ* ppm) *δ* 2.15 (s, 3H, CH_3_), 3.18 (s, 3H, N-CH_3_), 5.84 (s, 2H, CH_2_), 7.11 (s, 1H, C_3_-H), 7.18 (d, 1H, *J* = 8.8 Hz, Ar-H), 7.27–7.31 (m, 1H, Ar-H), 7.36–7.69 (m, 9H, Ar-H), 7.78 (d, 1H, *J* = 8 Hz, Ar-H), 7.82 (d, 1H, *J* = 9.2 Hz, Ar-H), 7.92 (d, 1H, *J* = 8 Hz, Ar-H), 7.98 (d, 1H, *J* = 9.2 Hz, Ar-H), 8.13 (d, 1H, *J* = 8.4 Hz, Ar-H), 9.22 (d, 1H, *J* = 8.4 Hz, Ar-H), 10.58 (s, 1H, -N = CH); ^13^C NMR (100 MHz, CDCl_3_, *δ* ppm): 10.50, 36.01, 71.67, 112.86, 113.77, 115.62, 118.06, 119.84, 121.17, 124.46, 124.81, 125.08, 125.72, 125.90, 126.88, 127.88, 128.35, 128.61, 129.29, 129.39, 130.02, 130.21, 131.41, 132.28, 132.37, 133.91, 134.91, 152.17, 152.73, 154.98, 155.60, 155.95, 160.39, 160.77; MS: 565[M]^+^; Anal. calcd for C_36_H_27_N_3_O_4_: C, 76.44; H, 4.81; N, 7.43%. Found: C, 76.34; H, 4.75; N, 7.38%.

#### 2.4.9. (E)-1,5-dimethyl-4-((2-((2-oxo-2H-benzo[h]chromen-4-yl)methoxy)naphthalen-1-yl) methyleneamino)-2-phenyl-1,2-dihydropyrazol-3-one **(3i)**

Yellow solid; Mp 202-204°C; IR (KBr) (*v*_max_ cm^−1^): 1727 (C = O of lactone), 3443 (NH stretching); ^1^H NMR (400 MHz, CDCl_3_, *δ* ppm) *δ* 2.17 (s, 3H, CH_3_), 3.19 (s, 3H, N-CH_3_), 5.84 (s, 2H, CH_2_), 7.11 (s, 1H, C_3_-H), 7.189 (d, 1H, *J* = 8.8 Hz, Ar-H), 7.27–7.33 (m, 1H, Ar-H), 7.38–7.69 (m, 9H, Ar-H), 7.78 (d, 1H, *J* = 8 Hz, Ar-H), 7.82 (d, 1H, *J* = 9.2 Hz, Ar-H), 7.92 (d, 1H, *J* = 8 Hz, Ar -H), 7.98 (d, 1H, *J* = 9.2 Hz, Ar-H), 8.13 (d, 1H, *J* = 8.4 Hz, Ar-H), 9.22 (d, 1H, *J* = 8.4 Hz, Ar-H), 10.57 (s, 1H, -N = CH); ^13^C NMR (100 MHz, CDCl_3_, *δ* ppm): 11.17, 36.01, 71.47, 113.10, 113.77, 115.62, 117.94, 119.84, 121.27, 124.34, 124.81, 125.08, 125.63, 125.90, 126.88, 127.88, 128.35, 128.61, 129.29, 129.39, 130.02, 130.21, 131.41, 132.28, 132.37, 133.78, 134.91, 152.17, 152.87, 154.98, 155.51, 156.04, 160.39, 160.77; MS: 565[M]^+^; Anal. calcd for C_36_H_27_N_3_O_4_: C, 76.44; H, 4.81; N, 7.43%. Found: C, 76.38; H, 4.72; N, 7.31%.

### 2.5. Biological assay methods

#### 2.5.1. Antibacterial activity

The newly synthesized compounds coumarin–dihydropyrimidine derivatives **(3a–3i)** were screened for *in vitro* antibacterial activity by agar-well diffusion method [34] against two Gram-positive (*Bacillus subtilis* (ATCC no. 23857) and *Staphylococcus aureus* (ATCC-12598)) and two Gram-negative (*Escherichia coli* (ATCC-25922) and *Pseudomonas aeruginosa* (ATCC No. 25619)) bacterial strains. In this experiment, antibiotic ciprofloxacin was used as reference standard to compare antibacterial activities. The synthesized compounds were dissolved in dimethyl sulfoxide (the stock solution 1 mg ml^−1^). Furthermore, the dilutions were prepared at the required quantities of 100, 50 and 25 µg ml^−1^ concentrations. To ensure that the solvent had no effect on bacterial growth, control test was also performed containing disc loaded with only DMSO at the same dilution used in our experiment. Test compound solutions prepared in DMSO were serially diluted and loaded (10 µl) to sterile filter paper discs (6 mm diameter), which finally contained (25, 50 and 100 µg ml^−1^) of the compound per disc, respectively. Impregnated discs were then dried for 1 h and placed on inoculated plates. The seeded plates were incubated at 37°C for 16 h. The radii of inhibition zones (in mm) were measured and the percentage inhibition of test compounds was related to the standard drug whose zone of inhibition was taken as 100%. The results of minimum inhibitory concentrations (MICs) of the synthesized compounds against bacterial species are determined.

#### 2.5.2. Anti-inflammatory activity

*Egg albumin denaturation method*. All the synthesized compounds were subjected to anti-inflammatory effect against denaturation of hen's egg albumin method [35] at the concentration (31.25 µg ml^−1^) with standard aceclofenac drug (31.25 µg ml^−1^). The mixture (5 ml) consisted of 0.2 ml of egg albumin (from fresh hen's egg), 2.8 ml of phosphate buffered saline (PBS, pH 6.4) and 2 ml of varying concentrations of coumarin-based pyrimidine compounds so that final concentrations become 31.25, 62.5, 125, 250, 500, 1000 µg ml^−1^ and similar volume of double-distilled water served as control. Then the mixtures were incubated at (37 ± 2)°C in an incubator (Bio-technics, India) for 15 min and then heated at 70°C for 5 min. After cooling, their absorbance was measured at 660 nm (SHIMADZU, UV-1800 Spectrophotometer) by using vehicle as blank. Aceclofenac sodium at the final concentration (31.25, 62.5, 125, 250, 500, 1000 µg ml^−1^) was used as reference drug and treated similarly for the determination of absorbance. The percentage inhibition of protein denaturation was calculated by using the following formula:

$$\%\text{ Inhibition}=100\times \frac{\text{Abs of control }-\text{ Abs of sample}}{\text{Abs of control}}.$$

### 2.6. Computational studies

For the docking of ligands to protein active sites and for estimating the binding affinities of docked compounds, Surflex-Dock module, a fully automatic docking tool available on Sybyl X-2.0 v., was used in this study.

#### 2.6.1. Docking simulations

The X-ray crystal structure of *S. aureus* dihydropteroate synthetase (PDB ID: 1AD4) enzyme [36] was obtained from the Protein Data Bank in PDB format as starting point. The synthesized compounds and the standard compounds tested in this study were docked to *S. aureus* dihydropteroate synthetase (PDB ID: 1AD4) enzyme using Surflex-Dock program in Sybyl-X software by incremental construction approach of building the structure in the active site so as to favour the binding affinity [37]. Finally, the docked ligands were ranked based on a variety of scoring functions that have been compiled into the single consensus score (C-score) [38].

## 3. Results and discussion

### 3.1. Chemistry

Synthesis of the Schiff base analogues (E)-4-((2-hydroxynaphthalen-1-yl)methyleneamino)-1,5-dimethyl-2-phenyl-1,2-dihydropyrazol-3-one **(1)** was carried out according to a convenient one-step procedure, that is, by the condensation of commercially available 4-aminoantipyrine and 2-hydroxy-1-naphthaldehyde in ethanol, which provided excellent yields [39]. The substituted 4-bromomethyl coumarins **(2a–2i)** were synthesized using a Pechman cyclization of the phenols with 4-bromoethylacetoacetate [40]. Condensation of the 4-bromomethyl coumarin **(2a–2i)** (0.0025 mol) with (E)-4-((2-hydroxynaphthalen-1-yl)methyleneamino)-1,5-dimethyl-2-phenyl-1,2-dihydropyrazol-3-one (**1**) (0.0025 mol) in anhydrous K_2_CO_3_ (0.00625 mol) using absolute acetone as the solvent at room temperature afforded substituted (E)-1,5-dimethyl-4-((2-((2-oxo-2H-chromen-4-yl)methoxy)naphthalen-1-yl)methyleneamino)-2-phenyl-1,2-dihydropyrazol-3-one **(3a–3i)** isolated by the usual work-up.

**Scheme 1.**
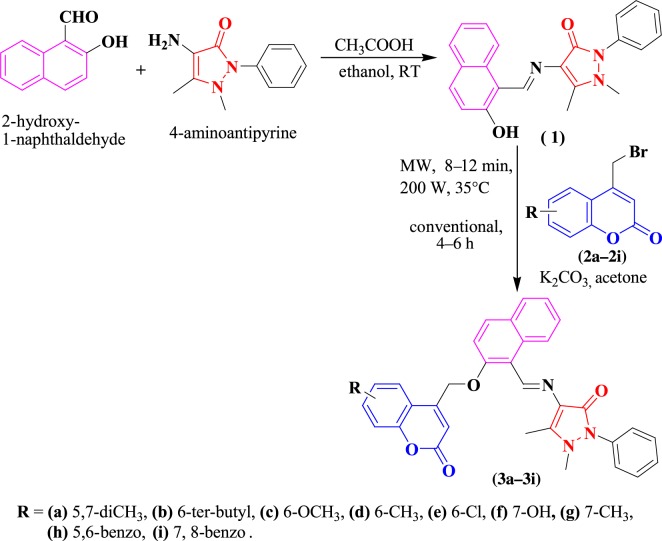
Synthetic route to synthesize the title compounds **(3a–3i)**.

Synthesis of coumarin derivatives was carried out under both conventional and microwave-irradiation methods. Synthesis of the target compounds was carried out as outlined in scheme 1. From the results, it is clear that the microwave approach proved to be extremely fast, providing good to excellent yields (82–95%) when compared with the conventional method (45–68%). Here, the most noticeable advancement was the speed with which the reactions were completed, i.e. within 8–12 min, which is 30–45 times faster than the conventional method. The results are summarized in table 1.

**Table 1.** Comparison between conventional and microwave irradiation method.

		yield (%)		time (min)	
products	*R*_1_	^a^C	^b^M	^a^C	^b^M
**3a**	5,7-diCH_3_	64	93	320	8
**3b**	6-ter-butyl	52	85	284	10
**3c**	6-OCH_3_	51	89	302	10
**3d**	6-CH_3_	68	91	256	8
**3e**	6-Cl	45	87	294	9
**3f**	7-OH	56	82	286	11
**3g**	7-CH_3_	62	95	240	12
**3h**	5,6-benzo	58	92	340	12
**3i**	7,8-benzo	49	88	360	9

^a^C, Conventional; ^b^M, Microwave.

All the newly synthesized compounds were characterized using FTIR, ^1^H NMR, ^13^C NMR, mass and elemental analysis. The physical and elemental analyses of all the compounds are given in Experimental section. The spectral data of the newly synthesized coumarin derivatives **(3a–3i)** are in accordance with the assigned structures of the compounds and are provided in the Experimental section. The ^1^H and ^13^C NMR spectra of all the compounds are given in the electronic supplementary material and are in good agreement with the proposed structure of the compounds.

In the case of compound **(3b)**, the IR spectrum exhibited two characteristic bands at 1724 cm^−1^ for lactone of the coumarin and 3444 cm^−1^ for NH stretching. The formation of the product was established using the ^1^H NMR spectrum (400 MHz, CDCl_3_) wherein a sharp singlet at δ 1.32 ppm corresponds to the tert-butyl of coumarin. Two singlets at δ 2.51 and 3.18 ppm correspond to the = C–CH_3_ and N–CH_3_ of the pyrazole. Furthermore, the presence of two singlet at δ 5.48 and 6.83 ppm corresponds to the CH_2_ and C_3_-H of the coumarin. One doublet was observed at δ 9.26 ppm with *J* = 8.4 Hz which corresponds to the Ar-H and a characteristic singlet for the imino proton (–CH = N–) at 10.57 ppm confirming the formation of the product.

The ^13^C NMR spectrum provides additional support for the structure of the compound **(3b)**, wherein the lactone carbonyl resonated at δ 156.34 ppm, pyrazole carbonyl resonated at δ 160.92 ppm and imino carbon resonated at δ 160.78 ppm. The two methyl carbons of N–CH_3_ and CH_3_ of pyrazole resonated at δ 36.06 and 10.51 ppm respectively. Tert-butyl and CH_2_ of coumarin resonated at δ 31.45 and 68.08 ppm, respectively. The molecular ion peak at 571 [M]^+^ in the mass spectrum confirmed the proposed structure of the compound **(3b)**. The rest of the compounds gave satisfactory analytical and spectroscopic data which were in accordance with their assigned structures.

The presence of the methylene protons approximately *δ* 5.2–5.4 ppm from derivatives led us to conclude that the initially formed ethers are stable and did not undergo a further intramolecular carbanion addition across the azomethine group located at close spatial proximity (ortho position) leading to the formation of 2,3-dihydronapthofurans by an intramolecular aldol addition followed by dehydration.

### 3.2. Computational studies

The newly synthesized compounds have exhibited excellent antibacterial activity, in particular against Gram-positive bacteria *S. aureus*. The *S. aureus* can cause a range of illnesses from minor skin infection to life-threatening diseases and has become resistant to many commonly used antibiotics. Ciprofloxacin is a synthetic chemotherapeutic antibiotic of the fluoroquinolone drug class and is a second-generation antibacterial agent, which kills bacteria by inhibiting the enzyme DNA-*gyrase*.

**Figure 4.**
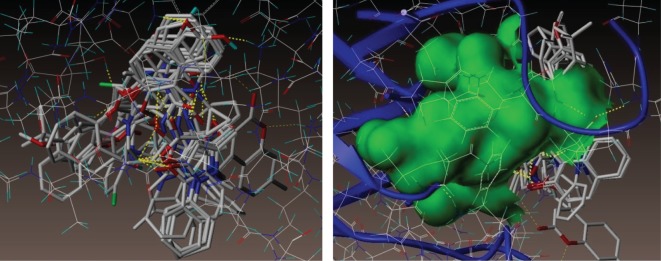
Docked view of all the synthesized compounds at the active site of the *S. aureus* enzyme (PDB ID: 1AD4).

To understand the mechanism of antibacterial activity of newly synthesized compounds, molecular modelling and docking studies were performed on X-ray crystal structure of the dihydropteroate synthetase (DHPS) complexed with OH-CH_2_-pterin-pyrophosphate from *S. aureus* (PDB ID: 1AD4, X-ray diffraction, 2.4 Å). Molecular docking was used to clarify the binding mode of the compounds to provide straightforward information for further structural optimization. The docking study was obtained from the Protein Data Bank by using Surflex-Dock program of Sybyl-X software. All the 10 compounds were docked into the active site of the DHPS (figure 4), the predicted binding energies and the observed C-score values of all the compounds are ranging from 4.27 to 10.33, the score values are listed in table 2.

**Table 2.** Surflex docking score (kcal mol^−1^) of the coumarin derivatives.

compounds	C-score^a^	crash score^b^	polar score^c^	D-score^d^	PMF score^e^	G-score^f^	Chem score^g^
**3a**	8.81	−3.91	3.79	−133.638	−88.498	−261.677	−35.782
**3b**	7.77	−3.63	2.42	−144.867	−91.593	−269.639	−34.697
**3c**	9.49	−1.85	4.38	−129.340	−96.820	−218.849	−36.228
**3d**	9.00	−2.58	3.45	−142.592	−94.903	−276.827	−44.811
**3e**	10.30	−4.16	4.39	−149.322	−101.748	−189.113	−38.703
**3f**	6.84	−1.18	1.82	−127.125	−50.500	−157.134	−20.523
**3g**	10.10	−3.91	3.90	−149.613	−83.797	−197.174	−36.644
**3h**	7.05	−6.13	2.60	−147.745	−49.089	−296.674	−32.790
**3i**	10.33	−0.89	1.84	−132.981	−76.170	−221.498	−31.537
ciprofloxacin	5.01	−2.26	1.91	−87.704	−69.857	−187.231	−21.485

^a^C-Score (consensus score) integrates a number of popular scoring functions for ranking the affinity of ligands bound to the active site of a receptor and reports the output of total score.

^b^Crash score revealing the inappropriate penetration into the binding site. Crash scores close to 0 are favourable. Negative numbers indicate penetration.

^c^Polar score indicating the contribution of the polar interactions to the total score. The polar score may be useful for excluding docking results that make no hydrogen bonds.

^d^D-score for charge and van der Waals interactions between the protein and the ligand.

^e^PMF score indicating the Helmholtz free energies of interactions for protein-ligand atom pairs (potential of mean force, PMF).

^f^G-score showing hydrogen bonding, complex (ligand–protein) and internal (ligand–ligand) energies.

^g^Chem score points for H-bonding, lipophilic contact and rotational entropy, along with an intercept term.

The proteins were prepared for docking by adding polar hydrogen atom with Gasteiger-Huckel charges and water molecules were removed. The 3D structure of the ligands was generated by the SKETCH module implemented in the SYBYL program (Tripos Inc., St. Louis, USA) and its energy-minimized conformation was obtained with the help of the Tripos force field using Gasteiger–Huckel [41] charges, molecular docking was performed with Surflex-Dock program that is interfaced with Sybyl-X2.0, and other miscellaneous parameters were assigned with the default values given by the software.

**Figure 5.**
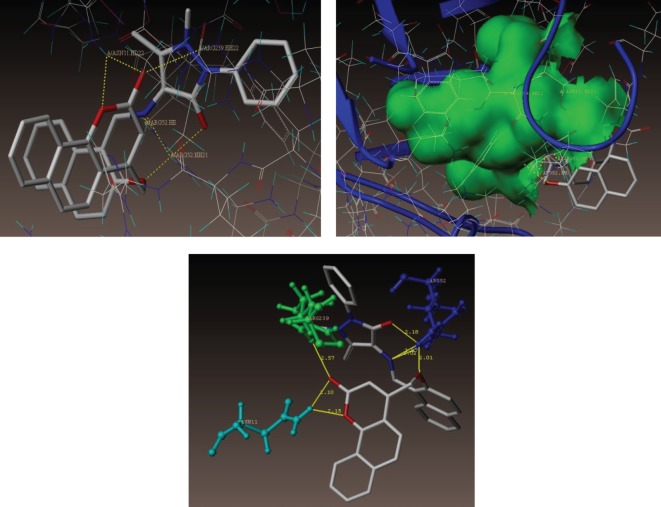
Docked view of the active site of the *S. aureus* subunit enzyme PDB: 1AD4 with compound **(3i)**, which shows the consensus score (C-score) of 10.33 and schematic of compound **(3i)** bound to the active site of the enzyme PDB: 1AD4 subunits.

As depicted in figure 5, compound **(3i)** makes seven hydrogen bonding interactions at the active site of the enzyme (PDB ID: 1AD4), among those two interactions were of nitrogen atom of C = N group with hydrogen of ARG52 (C = N------ H-ARG52, 2.02 Å, 2.07 Å), oxygen atom of carbonyl group present at coumarin ring makes hydrogen bonding interactions with hydrogens of ARG239 and ASN11 (C = O------H-ARG239, 2.57 Å; C = O------H-ASN11, 2.15 Å) oxygen atom of carbonyl group present at pyrazole ring makes hydrogen bonding interaction with hydrogen of ARG52 (C = O------H-ARG52, 2.18 Å) and remaining another hydrogen bonding interaction raised from the oxygen atom of CH_2_O group with hydrogen of ARG52 (CH_2_O ------H-ARG52, 2.01 Å).

**Figure 6.**
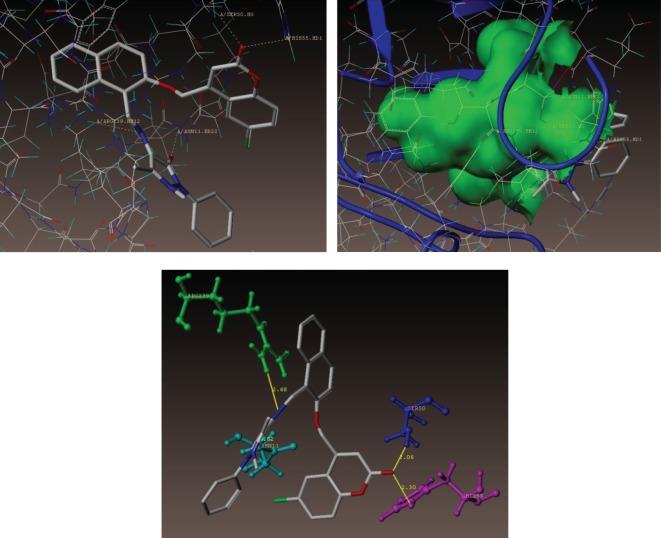
Docked view of the active site of the *S. aureus* subunit enzyme PDB: 1AD4 with compound **(3e)**, which shows the consensus score (C-score) of 10.30 and schematic of compound **(3e)** bound to the active site of the enzyme PDB: 1AD4 subunits.

As depicted in figure 6, compound **(3e)**, makes four hydrogen bonding interactions at the active site of the enzyme (PDB ID: 1AD4), among those, one interaction was of oxygen atom of carbonyl group present on the pyrazole ring with hydrogen of ASN11 (C = O------H-ASN11, 2.02 Å), nitrogen atom of C = N group makes an interaction with hydrogen of ARG239 (C = N------ H-ARG239, 2.68 Å), oxygen atom of carbonyl group present at coumarin ring makes hydrogen bonding interaction with hydrogen of HIS55 (C = O------H-HIS55, 2.30 Å) and oxygen atom of coumarin ring makes hydrogen bonding interaction with hydrogen of SER50 (C-O------H-SER50, 2.06 Å).

**Figure 7.**
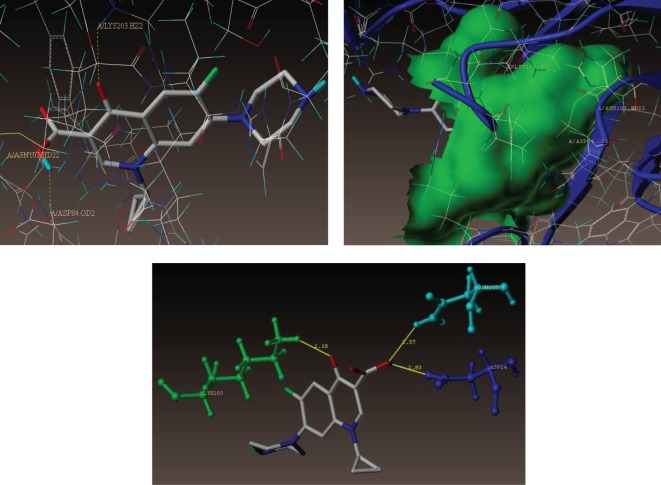
Interaction of ciprofloxacin at the binding site of the *S. aureus* subunit enzyme (PDB ID: 1AD4).

**Figure 8.**
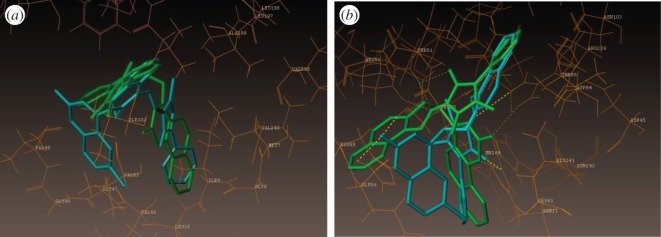
(*a*) Hydrophobic amino acids surrounded by compounds **(3i)** (green colour) and **(3e)** (cyan colour). (*b*) Hydrophilic amino acids surrounded by compounds **(3i)** and **(3e)**.

The binding interaction of standard ciprofloxacin with DHPS active sites shows three bonding interactions and the docked view of the same has been depicted in figure 7. The comparative molecular docking study of synthesized compounds and standard ciprofloxacin drug highlighted that the synthesized compounds exhibited high C-score value. C-score value (5.01) of ciprofloxacin was lower than those of all the nine compounds. Figure 8*a*,*b* represents the hydrophobic and hydrophilic amino acids surrounded by the studied compounds **(3i)** and **(3e).**

### 3.3. Pharmacological screening

It is evident from the above fact that compounds possessing coumarin and antipyrine moiety are capable of exhibiting biological activities. When this ring system is fused or coupled with other heterocycles, the resulting compounds would exhibit enhanced biological properties. Hence, novel coumarin–antipyrine derivatives **(3a–3i)** have been synthesized in the course of present investigation. These compounds have been screened for their potential *in vitro* antibacterial activity by agar-well diffusion method and anti-inflammatory activity by egg albumin denaturation method.

#### 3.3.1. *In vitro* antibacterial screening

**Figure 9.**
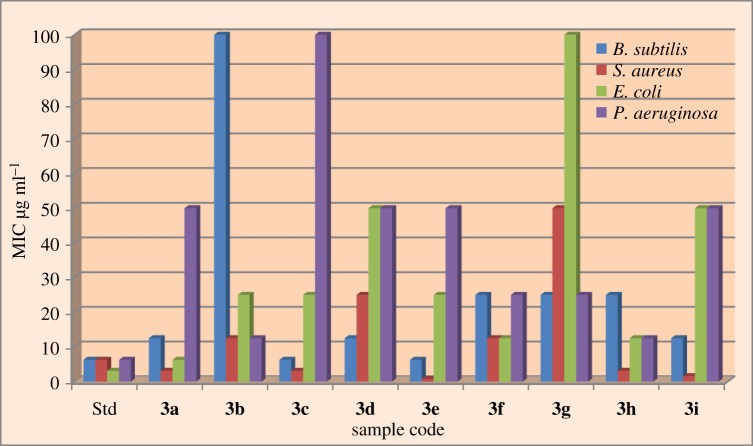
Graphical representation of minimum inhibitory concentrations (MIC) (µg ml^−1^) of all the compounds against *B. subtilis, S. aureus, E. coli* and *P. aeruginosa*.

The newly synthesized compounds were screened for *in vitro* antibacterial activity by agar-well diffusion method [34] against two Gram-positive (*Bacillus subtilis* (ATCC no. 23857) *and Staphylococcus aureus* (ATCC-29213)) and two Gram-negative (*Escherichia coli* (ATCC-25922) and *P. aeruginosa* (ATCC No. 25619)) bacterial strains. The minimum inhibitory concentration (MIC) of the synthesized compounds **(3a–3i)** and ciprofloxacin was compared, it revealed that almost all the newly synthesized compounds showed excellent antibacterial activity against Gram-positive *S. aureus* bacterial strain. Screening results are summarized in table 3. The best antibacterial effect has compounds **(3e)** and **(3i)** with MIC 0.78 µg ml^−1^ and MIC 1.562 µg ml^−1^, respectively, against Gram-positive *S. aureus* bacterial strain*.* Similarly compounds **(3a)** and **(3c)** showed better activity against Gram-positive and Gram-negative bacteria. While remaining compounds **(3b, 3d, 3f, 3g, 3i)** lead to weak antibacterial activity. The results are also represented in bar diagram of figure 9.

**Table 3.** *In vitro* antibacterial screening for compounds **(3a–3i)**.

		microorganisms used for antibacterial activity (MIC µg ml^−1^)			
		Gram positive		Gram negative	
products	*R*	*B. subtilis*	*S. aureus*	*E. coli*	*P. aeruginosa*
**3a**	5,7-diCH_3_	12.5	3.125	6.25	50
**3b**	6-ter-butyl	100	12.5	25	12.5
**3c**	6-OCH_3_	6.25	3.125	25	100
**3d**	6-CH_3_	12.5	25	50	50
**3e**	6-Cl	6.25	0.78	25	50
**3f**	7-OH	25	12.5	12.5	25
**3g**	7-CH_3_	25	50	100	25
**3h**	5,6-benzo	25	3.125	12.5	12.5
**3i**	7,8-benzo	12.5	1.562	50	50
ciprofloxacin^a^		6.25	6.25	3.125	6.25

^a^Ciprofloxacin was used as a positive control against bacteria species.

#### 3.3.2. *In vitro* anti-inflammatory activity

**Figure 10.**
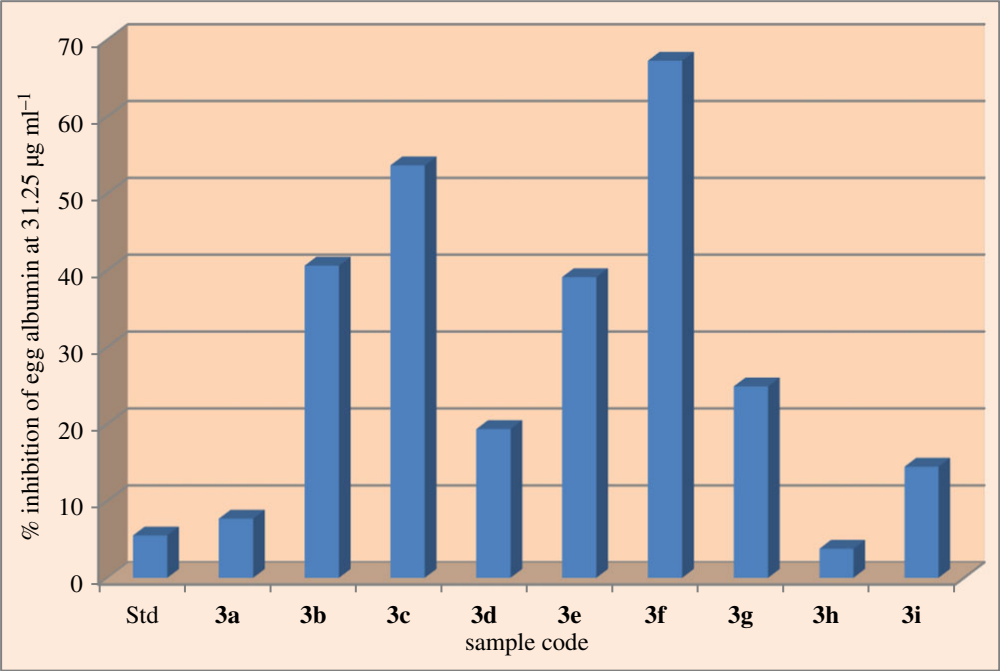
Graphical representation of % inhibition of egg albumin in 31.25 µg ml^−1^ of all the compounds **(3a–3i).**

The outcome of anti-inflammatory screening of compounds **(3a–3j)**, by using egg albumin denaturation method, is summarized in table 4. The percentage inhibition of all the synthesized compounds was very highly active against the denaturation of protein. Among these compounds, **(3c)** and **(3f)** exhibited an excellent inhibition of heat-induced protein denaturation 53.65% and 67.27%, respectively, and these compounds are almost 10 times more active than standard aceclofenac drug (5.50%). Whereas compounds **(3a)** and **(3h)** show less activity and the remaining compounds showed good inhibitory anti-inflammatory activity against the denaturation of protein method. The synthesized compounds have shown significant anti-inflammatory activity in protein denaturation method. The results are also represented in the bar diagram of figure 10. These results suggest that an electron releasing group (–OCH_3_) and hydrogen bonding group (–OH) increases the anti-inflammatory potency.

**Table 4.** *In vitro* anti-inflammatory activity in protein denaturation method of compounds **(3a–3i)**. Values are mean ± s.d., *n* = 3.

entry	*R*	% inhibition of egg albumin in 31.25 µg ml^−1^
control	—	—
**3a**	5,7-diCH_3_	7.69 ± 0.03
**3b**	6-ter-butyl	40.59 ± 0.01
**3c**	6-OCH_3_	53.65 ± 0.03
**3d**	6-CH_3_	19.33 ± 0.04
**3e**	6-Cl	39.09 ± 0.03
**3f**	7-OH	67.27 ± 0.05
**3g**	7-CH_3_	24.87 ± 0.01
**3h**	5,6-benzo	3.78 ± 0.05
**3i**	7,8-benzo	14.44 ± 0.02
aceclofenac	—	5.50 ± 0.01

### 3.4. Structural activity relationship study

Even though the number of compounds tested here is limited, a few key features regarding structural requirements for these coumarin-pyrazole hybrids **(3a–3i)** to exert their antibacterial activity may be determined. Our initial strategy was to identify the key subunity required for activity such as coumarin (antibacterial agents, which allows its derivatives to readily interact with diversity of enzymes and receptors in organisms), pyrazole (nitrogen heterocyclic-active pharmacophore) and naphthalene group fused to coumarin (lipid-lowering agents and photo-physical character). Further essential substituents like [**R** = -CH_3_ and –OCH_3_] (electron donating) groups were varied at 6 and 7-position of the coumarin ring to get the optimum results. The designed hypothetical interaction module is represented in figure 3.

The results demonstrated the following assumptions about the structural activity relationship (SAR): compound **(3e)** having –Cl substituent at 6-position of coumarin was found to be most active against Gram-positive *S. aureus* bacterial strain exhibiting MIC of 0.78 µg ml^−1^. Benzo group when attached to coumarin at 7,8-position **(3i)** enhances the activity with MIC of 1.562 µg ml^−1^. The change in the position of benzo group to 5,6-position **(3h)** slightly lowers the activity with MIC of 3.125 µg ml^−1^. Electron-donating -CH_3_ groups, when present at 6-position of coumarin **(3d),** exhibit MIC of 25 µg ml^−1^ and, at 7-position of coumarin **(3g),** exhibit MIC of 50 µg ml^−1^ but when both –CH_3_ groups are present at 5 and 7-position **(3a)** exhibit increased antibacterial activity with MIC of 3.125 µg ml^−1^. The results from the preliminary structure-activity analysis have led to the determination of some key structural requirements for the coumarin-pyrazole hybrids to exert their antibacterial activity, which provide insights into further structural modification.

## 4. Conclusion

To explore different scaffold structures, we have described environmentally benign, simple and efficient protocol for the synthesis of coumarin-based pyrazole derivatives **(3a–3i)** with high yields under microwave-irradiation in shorter reaction time (8–12 min), being 30–45 times faster than the conventional method. Antibacterial screening revealed that compounds **(3e)** and **(3i)** exhibited potent activity against *S. aureus* bacterial strain with MIC 0.78 µg ml^−1^ and 1.562 µg ml^−1^, respectively. The compound **(3f)** exhibited an inhibition of heat-induced protein denaturation at the concentration (31.25 µg ml^−1^) as 67.27%. Among all these synthesized scaffolds, compounds **(3e)** and **(3i)** are highly active and more potent in both biological and molecular docking simulation studies. The biological activities of coumarins after combining with pyrazole have been enhanced due to synergistic effect. The results also suggested a new and potential route in the discovery of drug against antibacterial and anti-inflammatory activities.

## Supplementary Material

Supplementary data

## Data accessibility

This article does not contain any additional data.
