# Supplementary material for: Microwave-assisted synthesis, computational studies and antibacterial/ anti-inflammatory activities of compounds based on coumarin-pyrazole hybrid
Source: R Soc Open Sci. 2018 May 2;5(5):172435. doi: 10.1098/rsos.172435 (PMC5990750; doi:10.1098/rsos.172435)
Supplement: Supplementary data [file rsos172435supp1.docx]

**Microwave-assisted synthesis, computational studies and antibacterial / anti-inflammatory activities of compounds based on coumarin-pyrazole hybrid**

Rakesh R. Chavan and Kallappa M. Hosamani*

Department of Studies in Chemistry, Karnatak University, Dharwad -580003, India.

__________________________________________________________________________

***Corresponding authors**. Tel.: 09448422313; Fax: 0836 2747884 & 0836 2446601

E-mail: dr_hosamani@yahoo.com

**Supporting Information**

| **Contents** | **Page No** |
| --- | --- |
| ^1^H NMR, ^13^C NMR, IR, and Mass spectra of synthesized compounds | S2 – S19 |


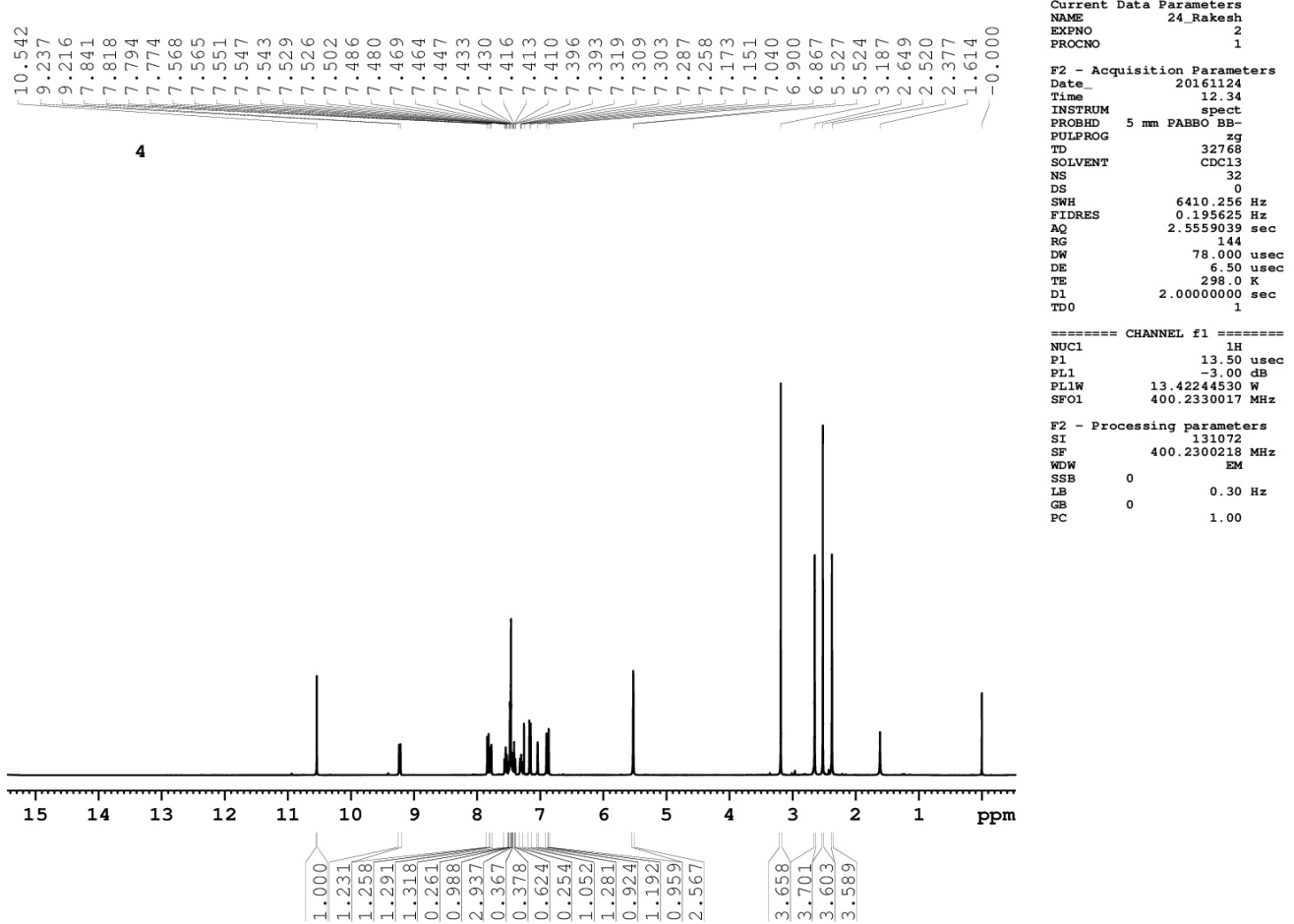
**Spectrum 1:** ^1^H NMR Spectrum of compound **(3a)** in DMSO-d_6_


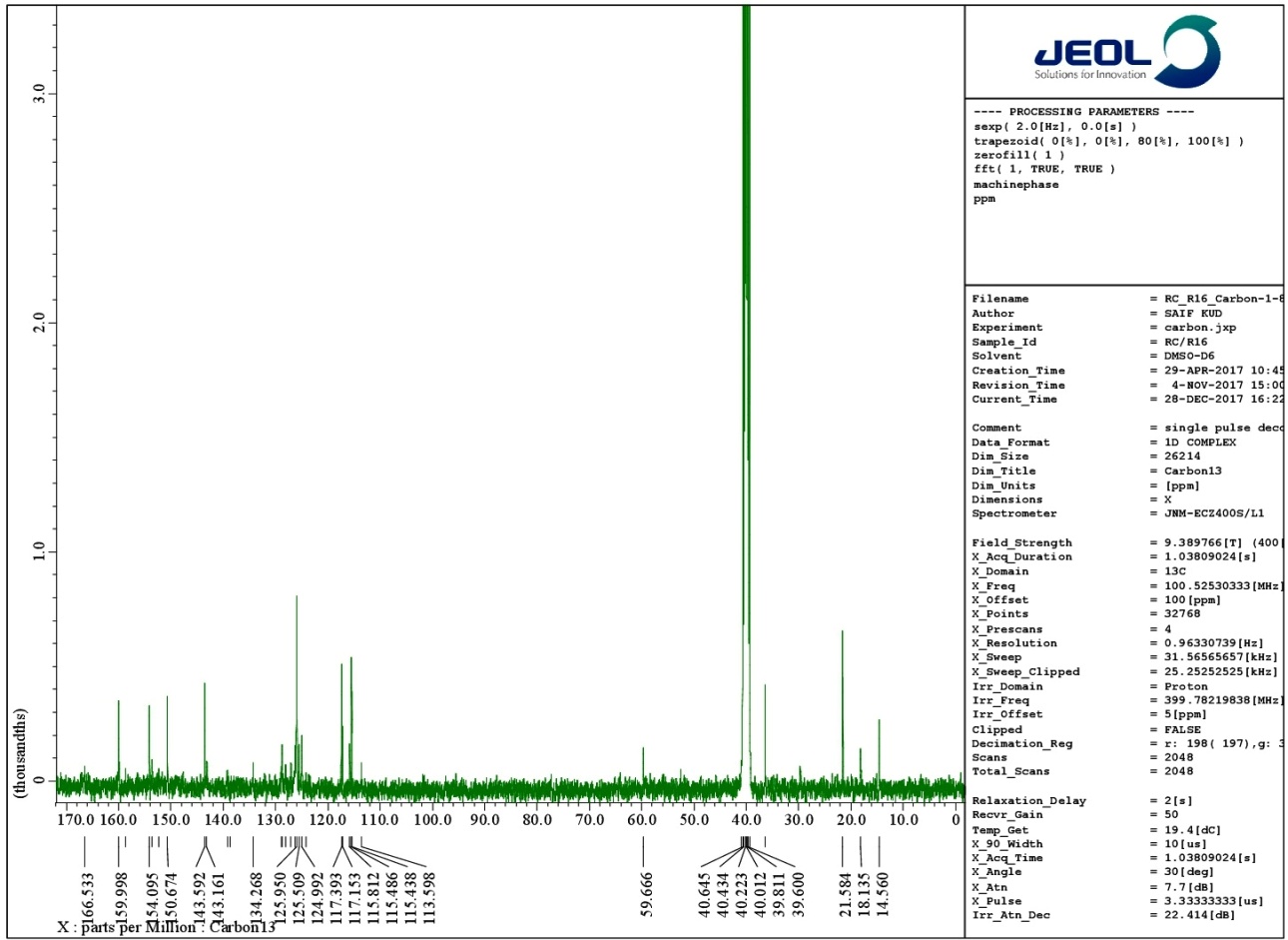


**Spectrum 2:** ^13^C NMR Spectrum of compound **(3a)** in DMSO-d_6_


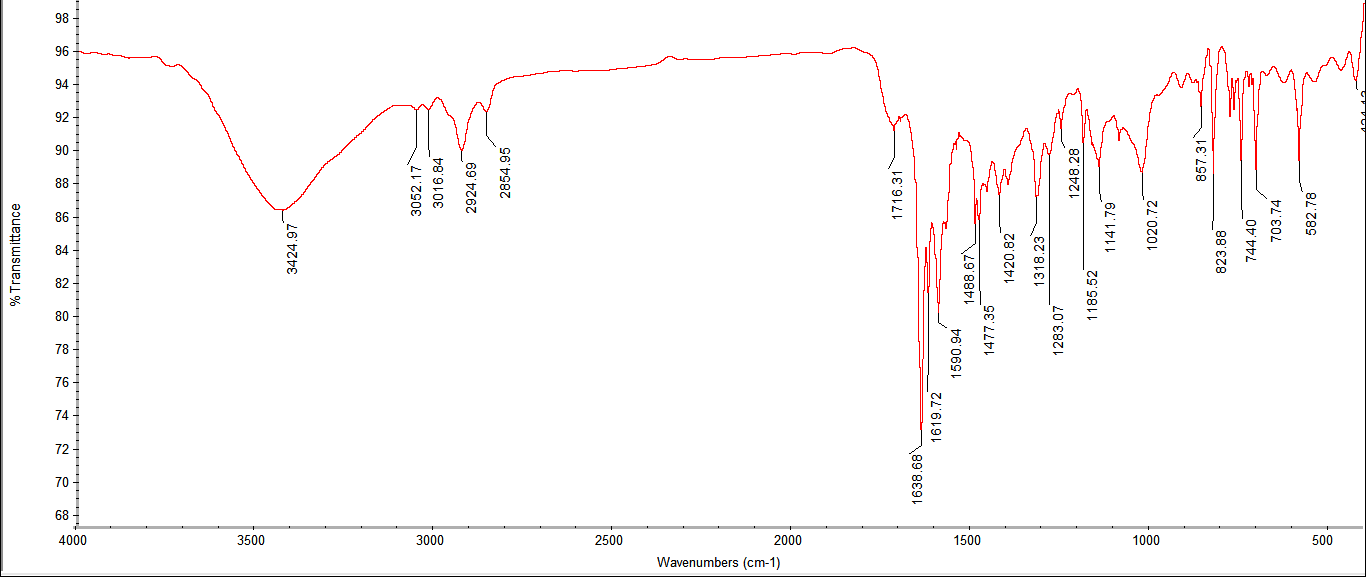


**Spectrum 3:** IR Spectrum of compound **(3a)**

**
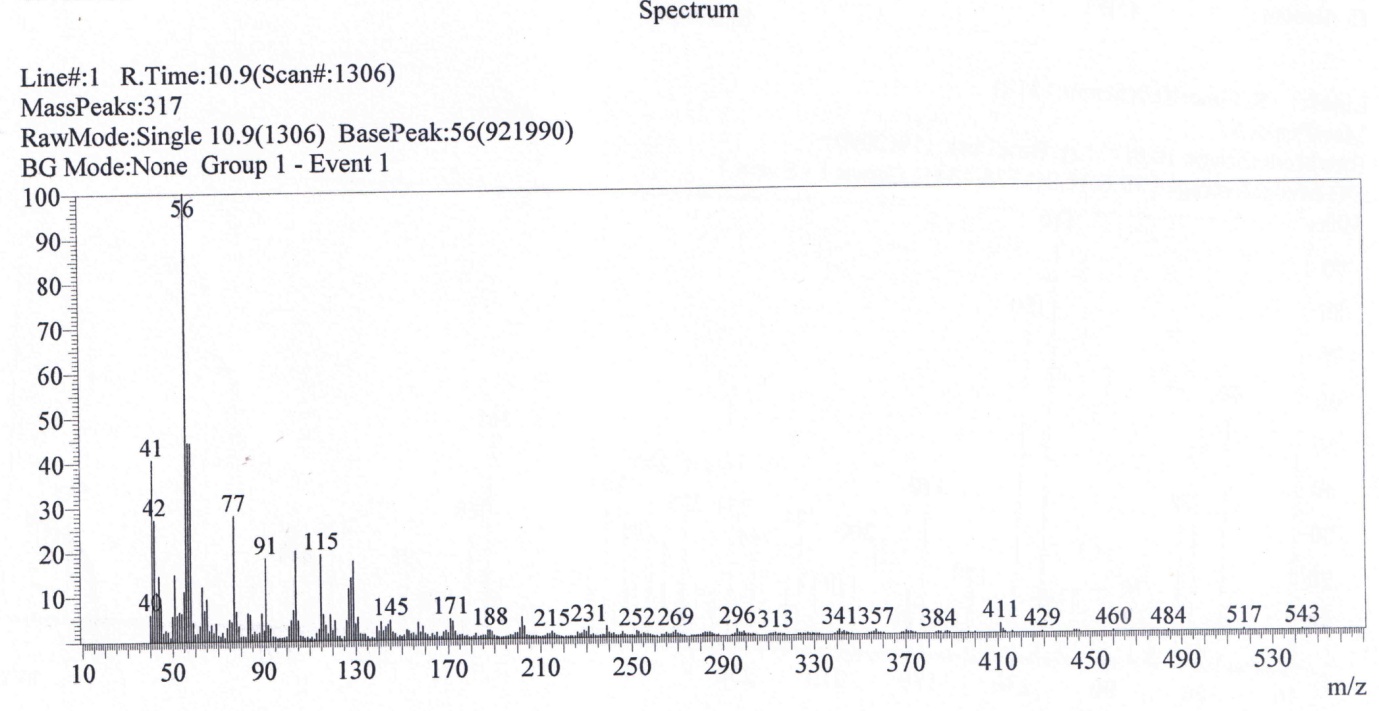
**

**Spectrum 4:** Mass Spectrum of compound **(3a)**

**
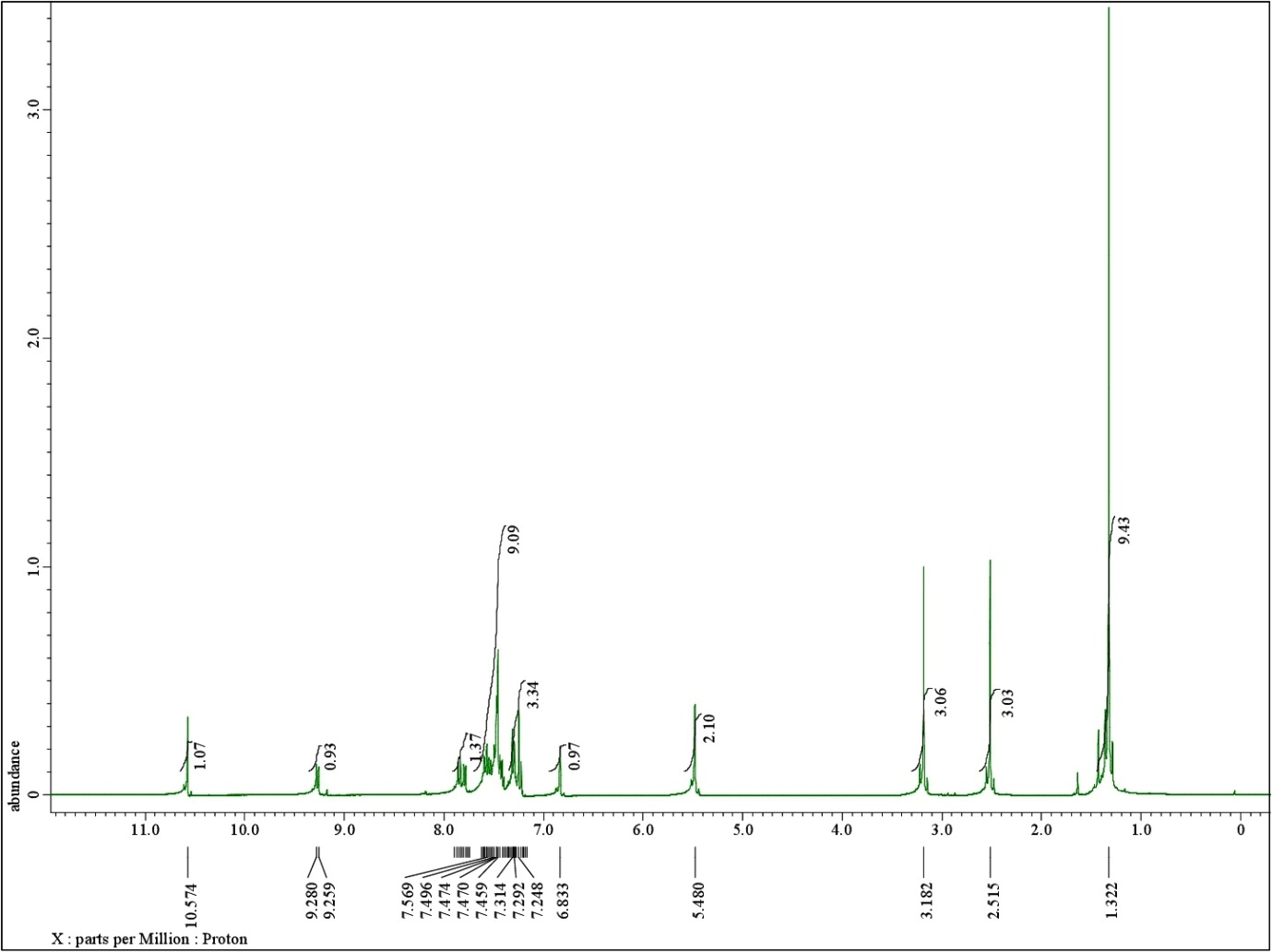
**

**Spectrum 5:** ^1^H NMR Spectrum of compound **(3b)** in DMSO-d_6_


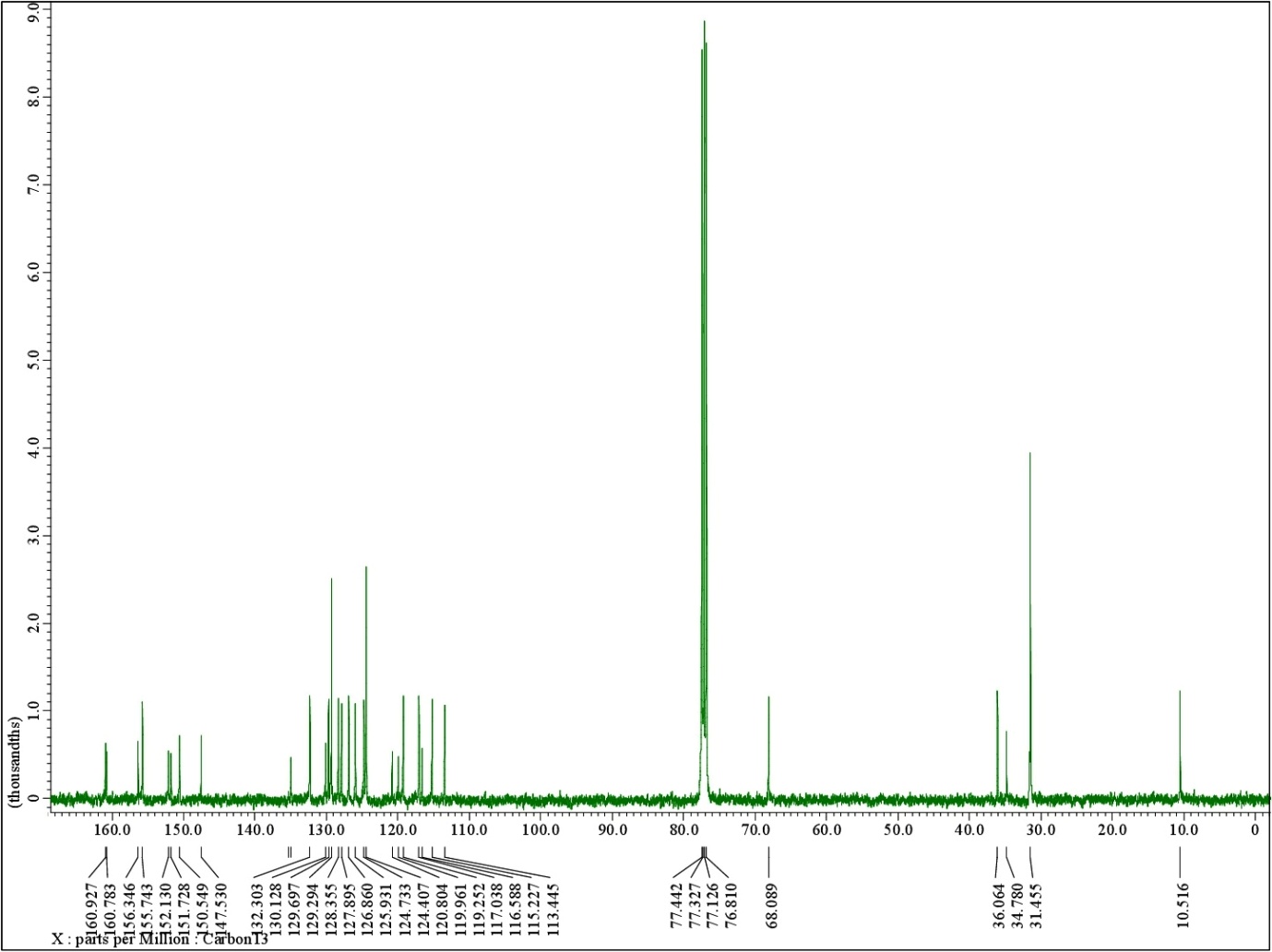


**Spectrum 6:** ^13^C NMR Spectrum of compound **(3b)** in DMSO-d_6_


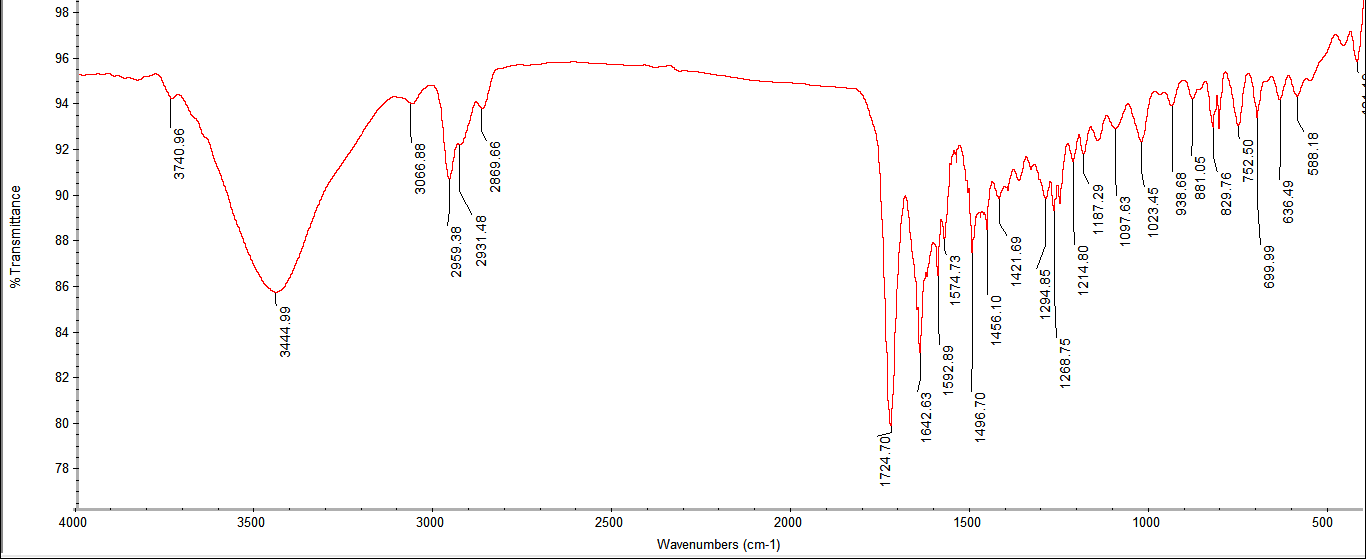


**Spectrum 7:** IR Spectrum of compound **(3b)**


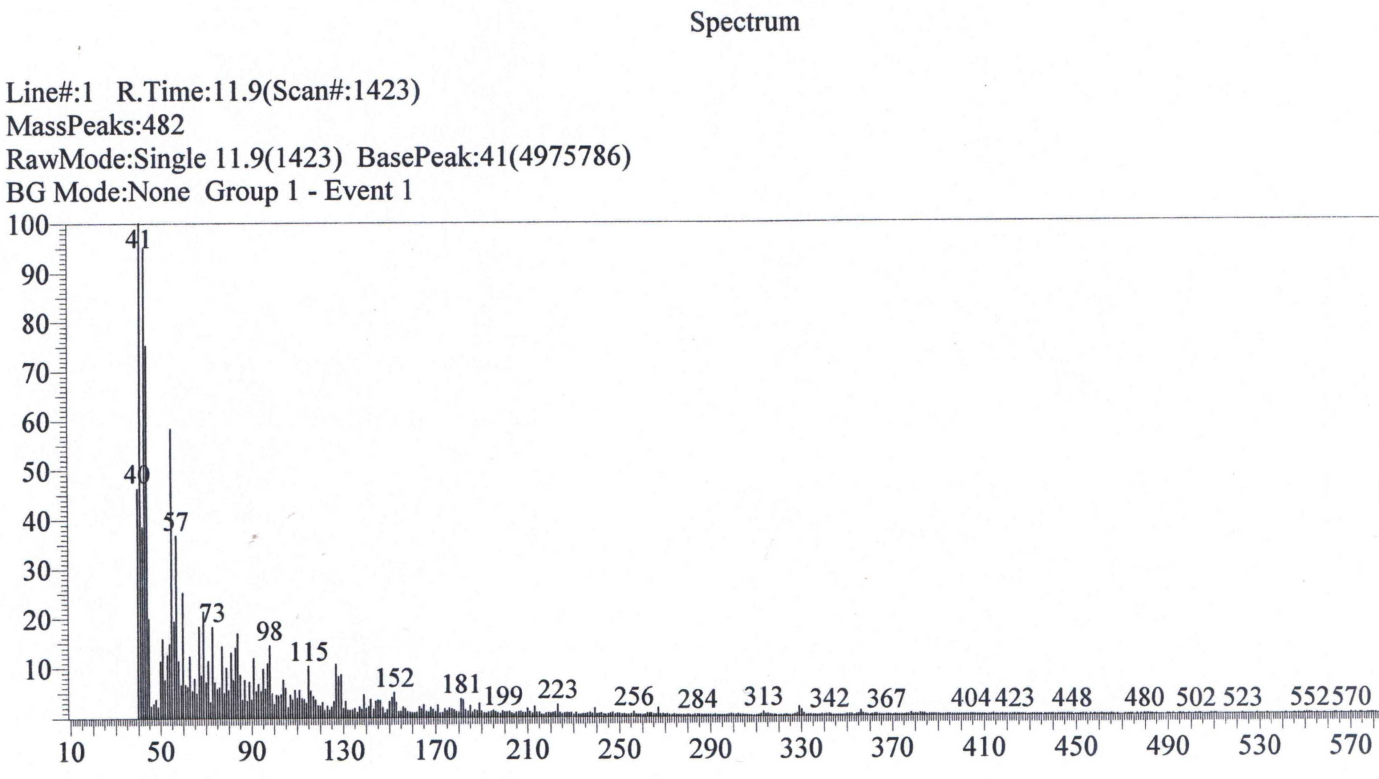


**Spectrum 8:** Mass Spectrum of compound **(3b)**

**
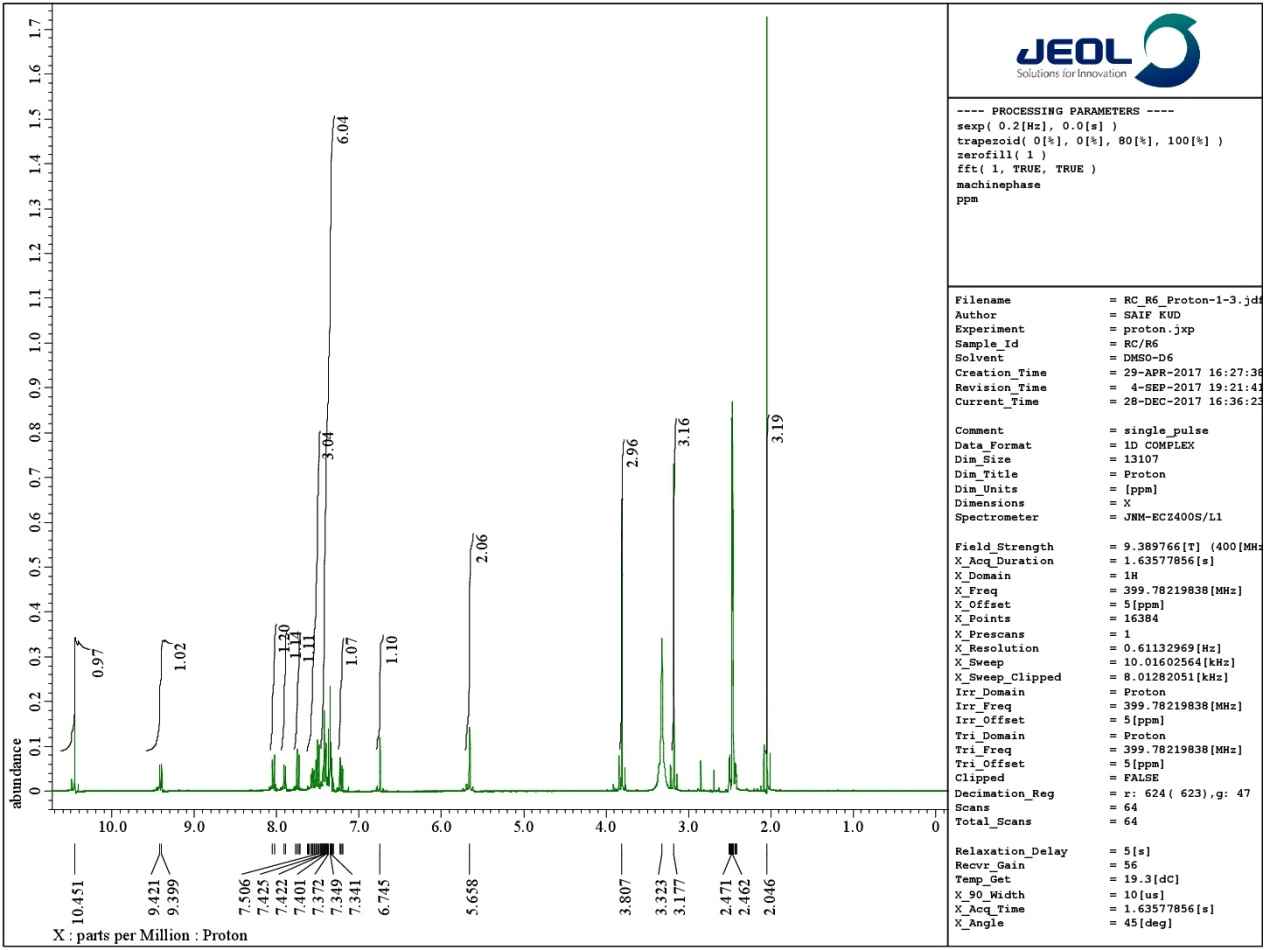
**

**Spectrum 9:** ^1^H NMR Spectrum of compound **(3c)** in DMSO-d_6_

**
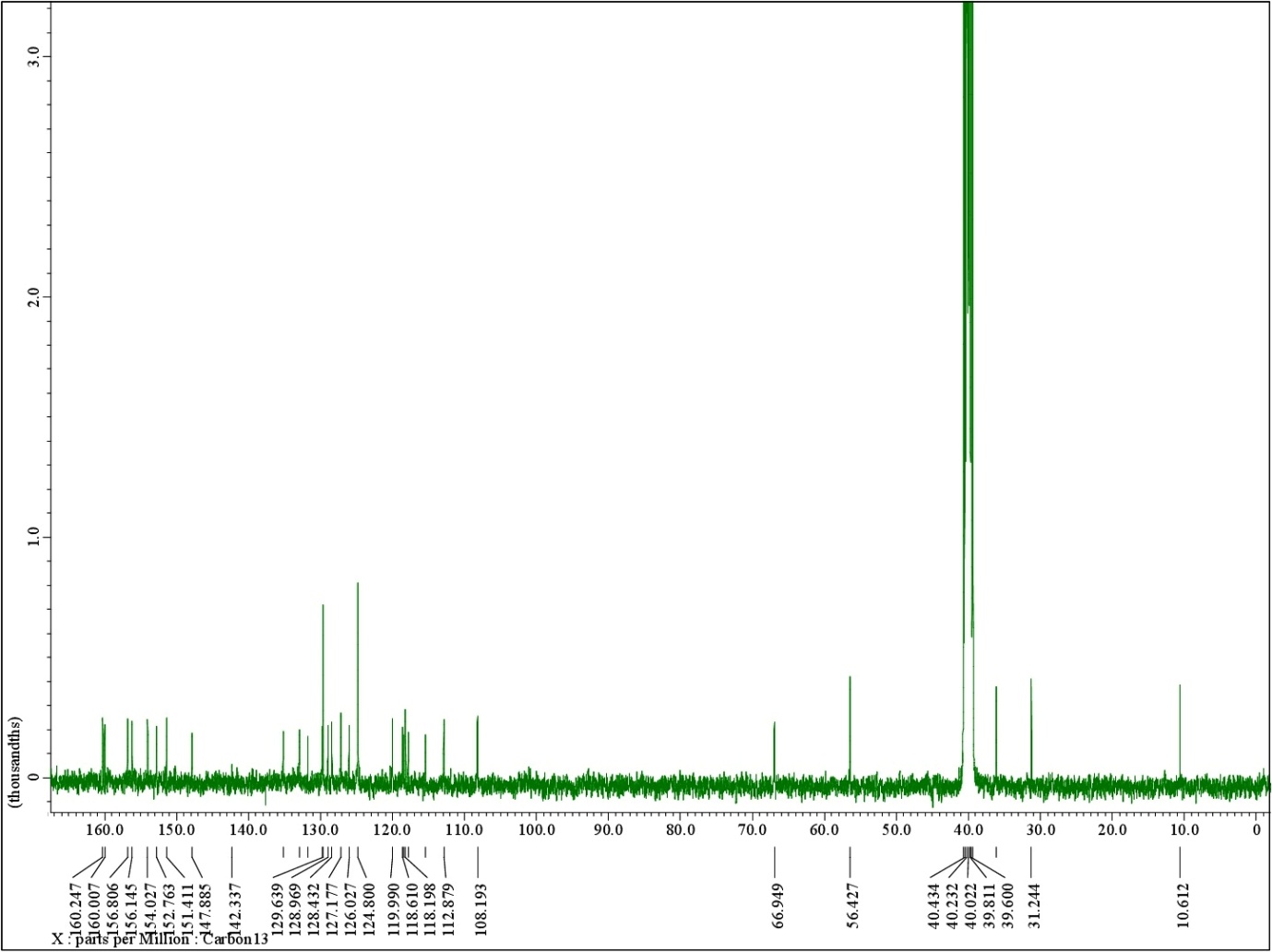
**

**Spectrum 10:** ^13^C NMR Spectrum of compound **(3c)** in DMSO-d_6_

**
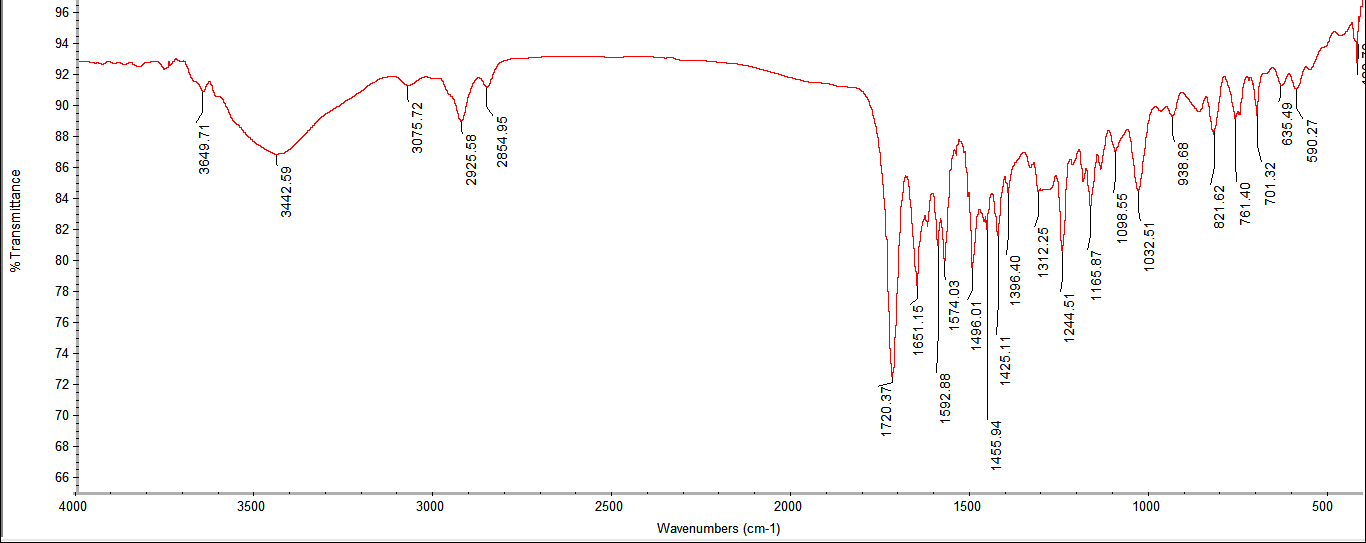
**

**Spectrum 11:** IR Spectrum of compound **(3c)**

**
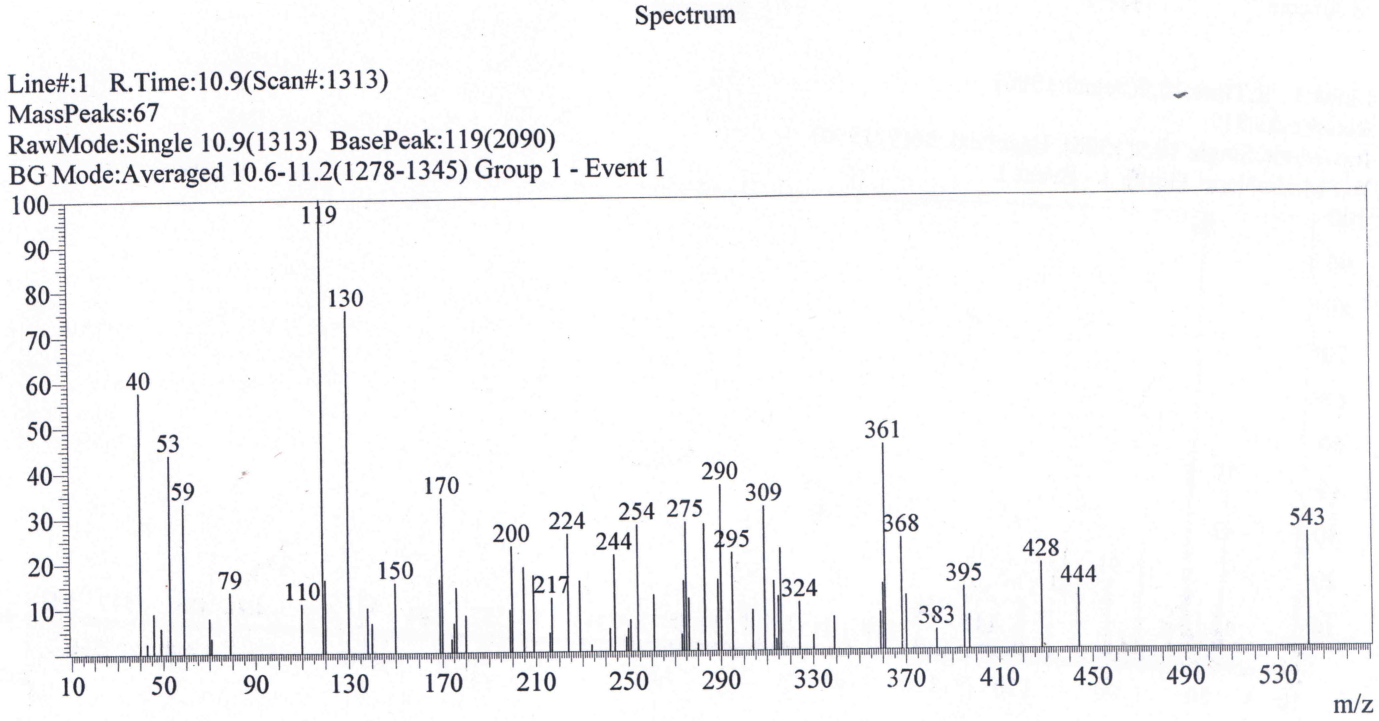
**

**Spectrum 12:** Mass Spectrum of compound **(3c)**

**
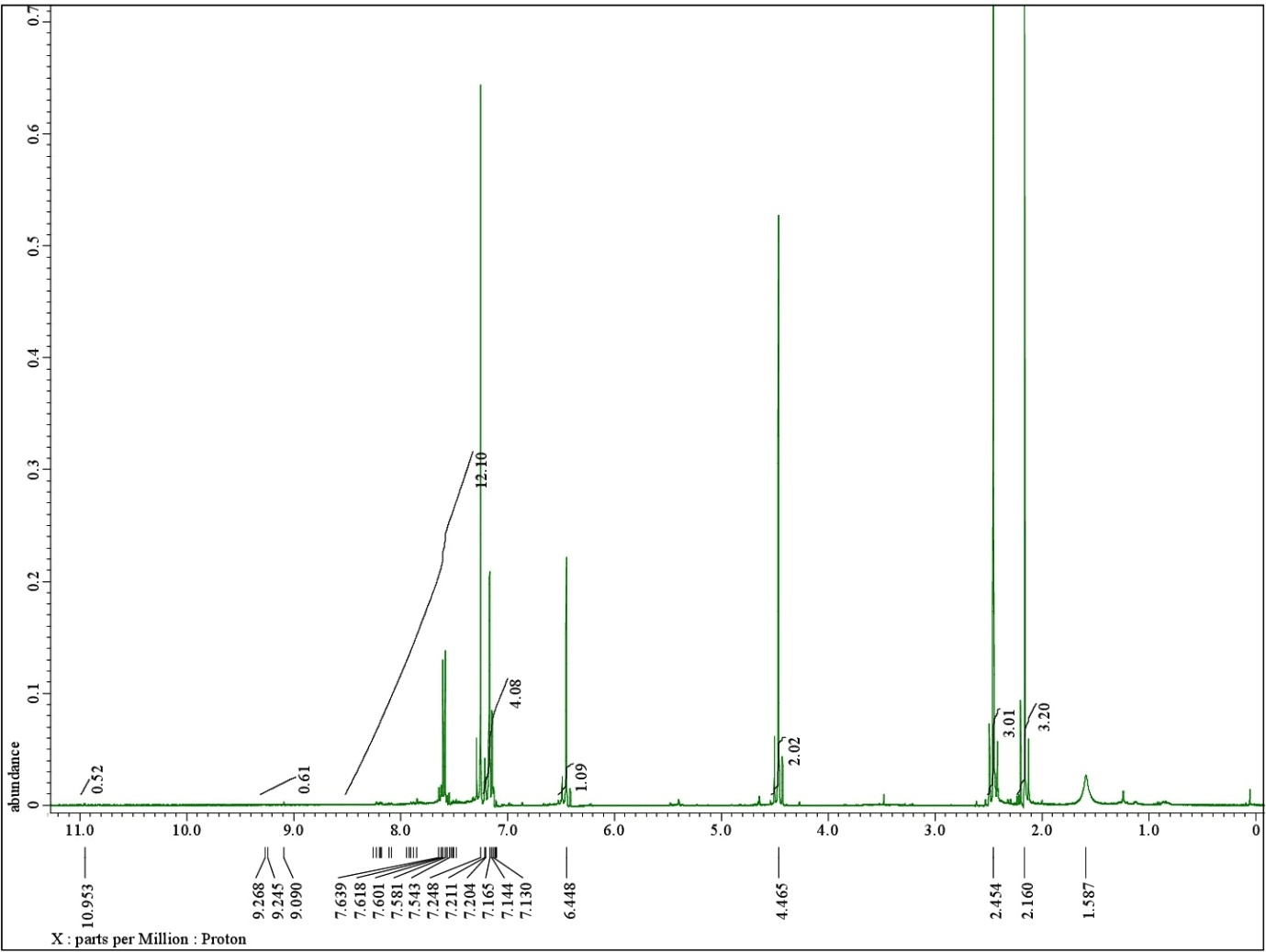
**

**Spectrum 13:** ^1^H NMR Spectrum of compound **(3d)** in DMSO-d_6_


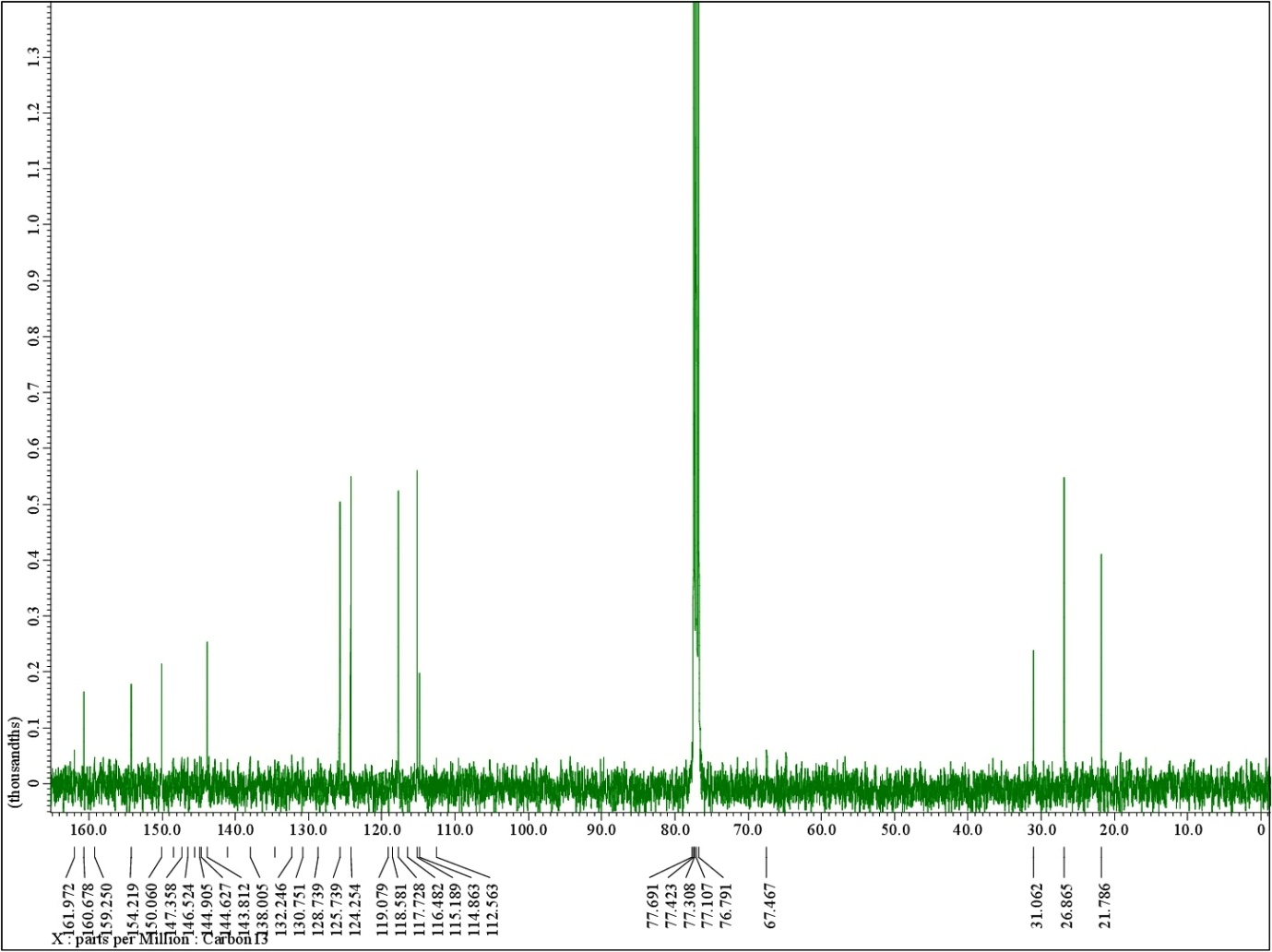


**Spectrum 14:** ^13^C NMR Spectrum of compound **(3d)** in DMSO-d_6_


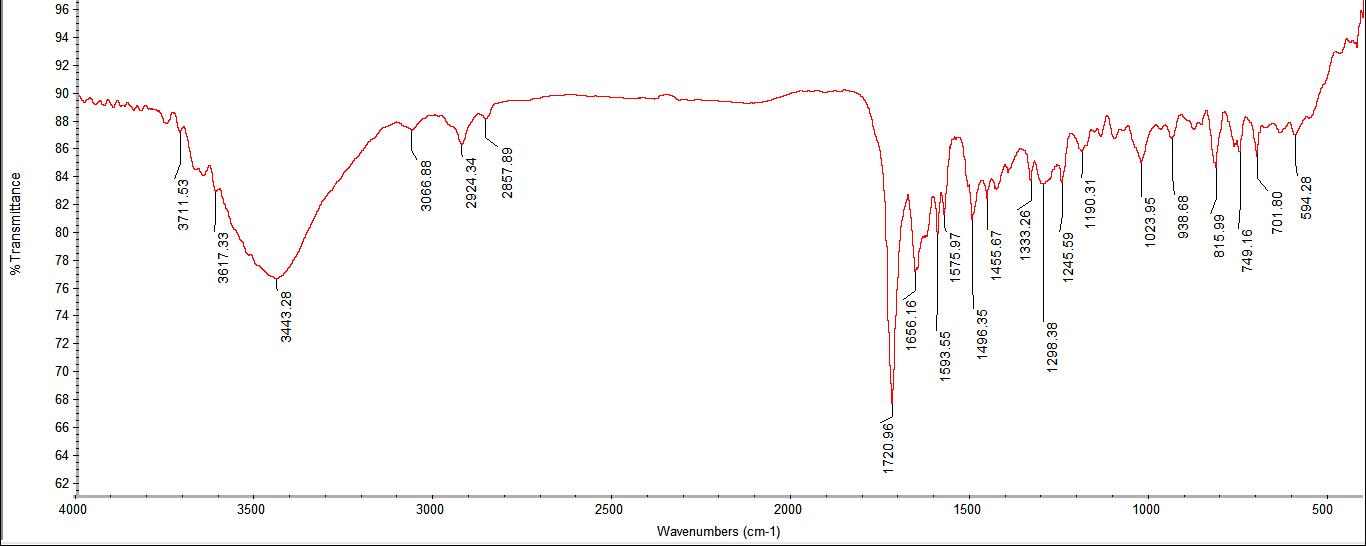


**Spectrum 15:** IR Spectrum of compound **(3d)**

**
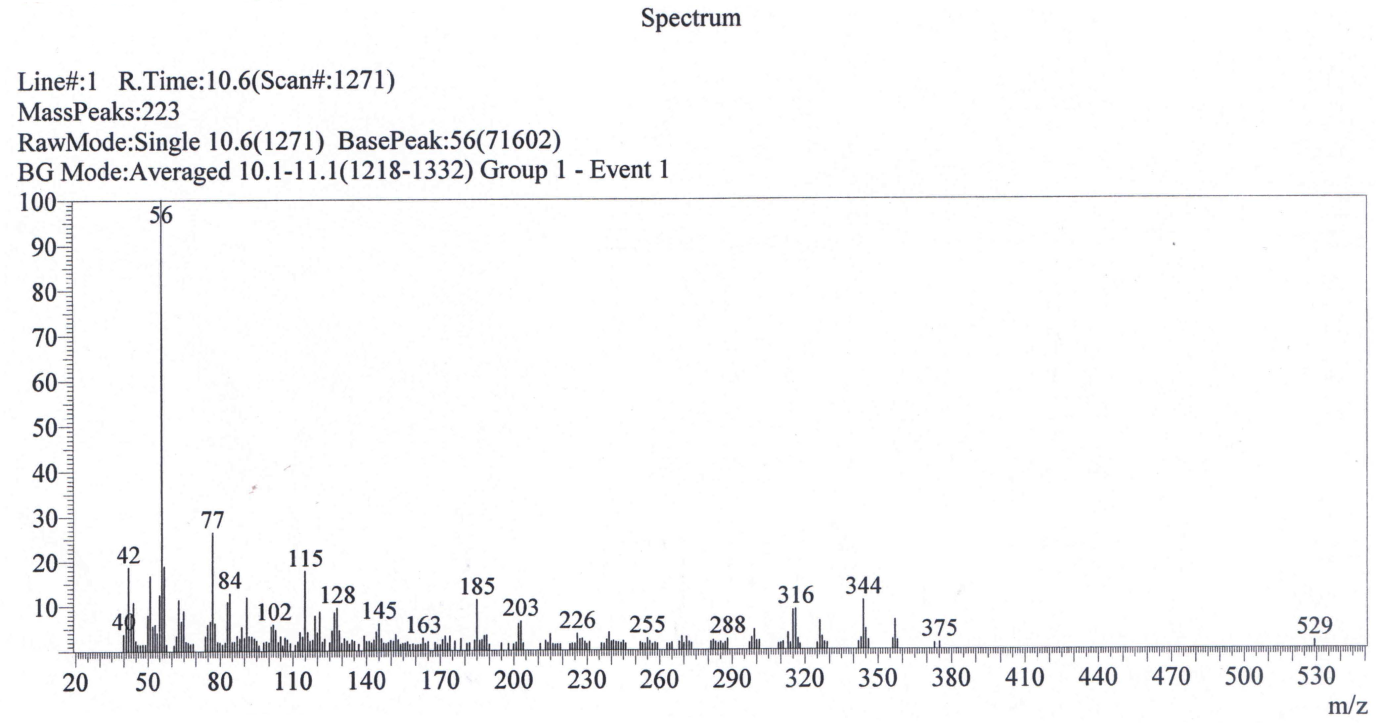
**

**Spectrum 16:** Mass Spectrum of compound **(3d)**


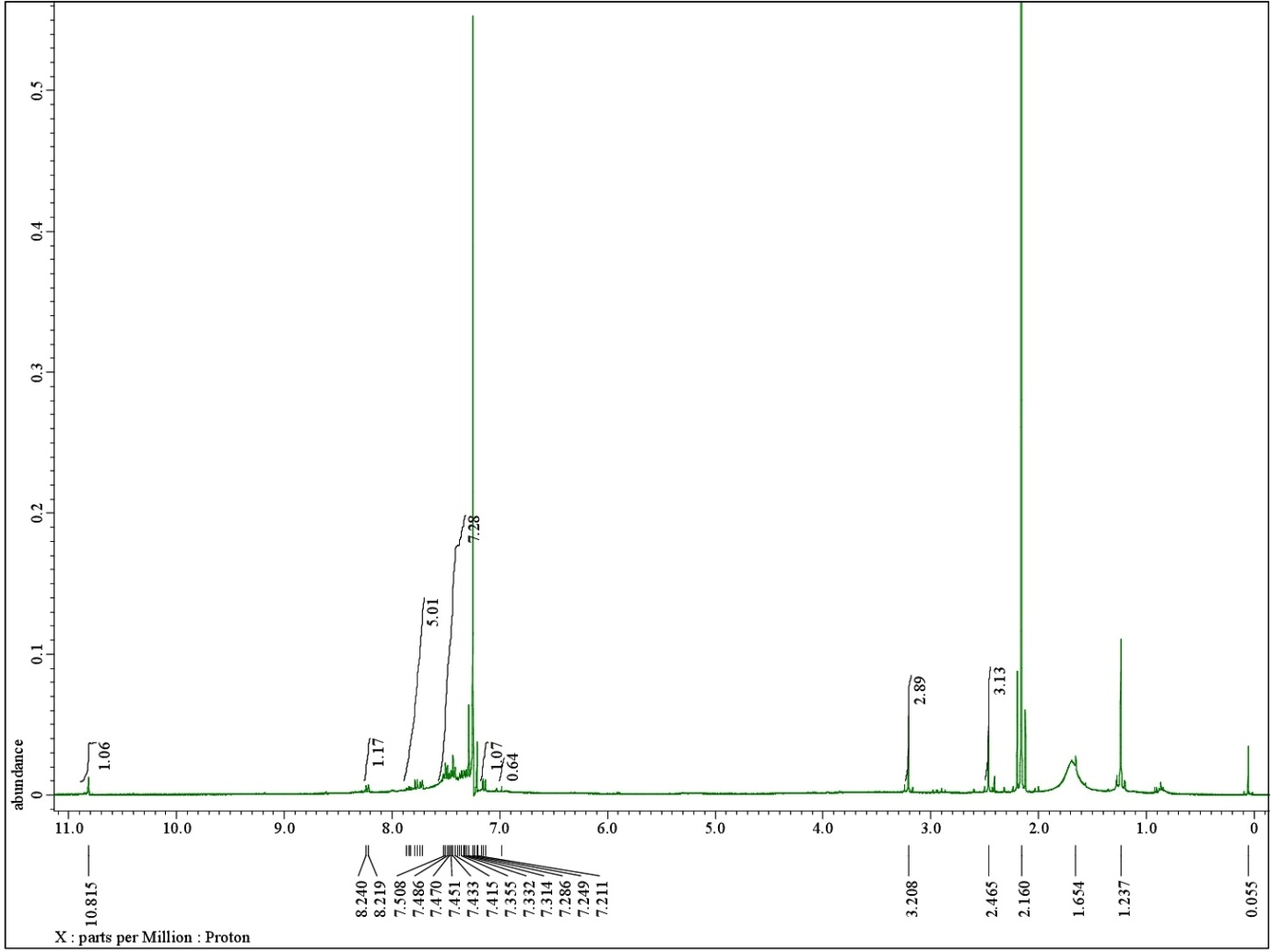


**Spectrum 17:** ^1^H NMR Spectrum of compound **(3e)** in DMSO-d_6_


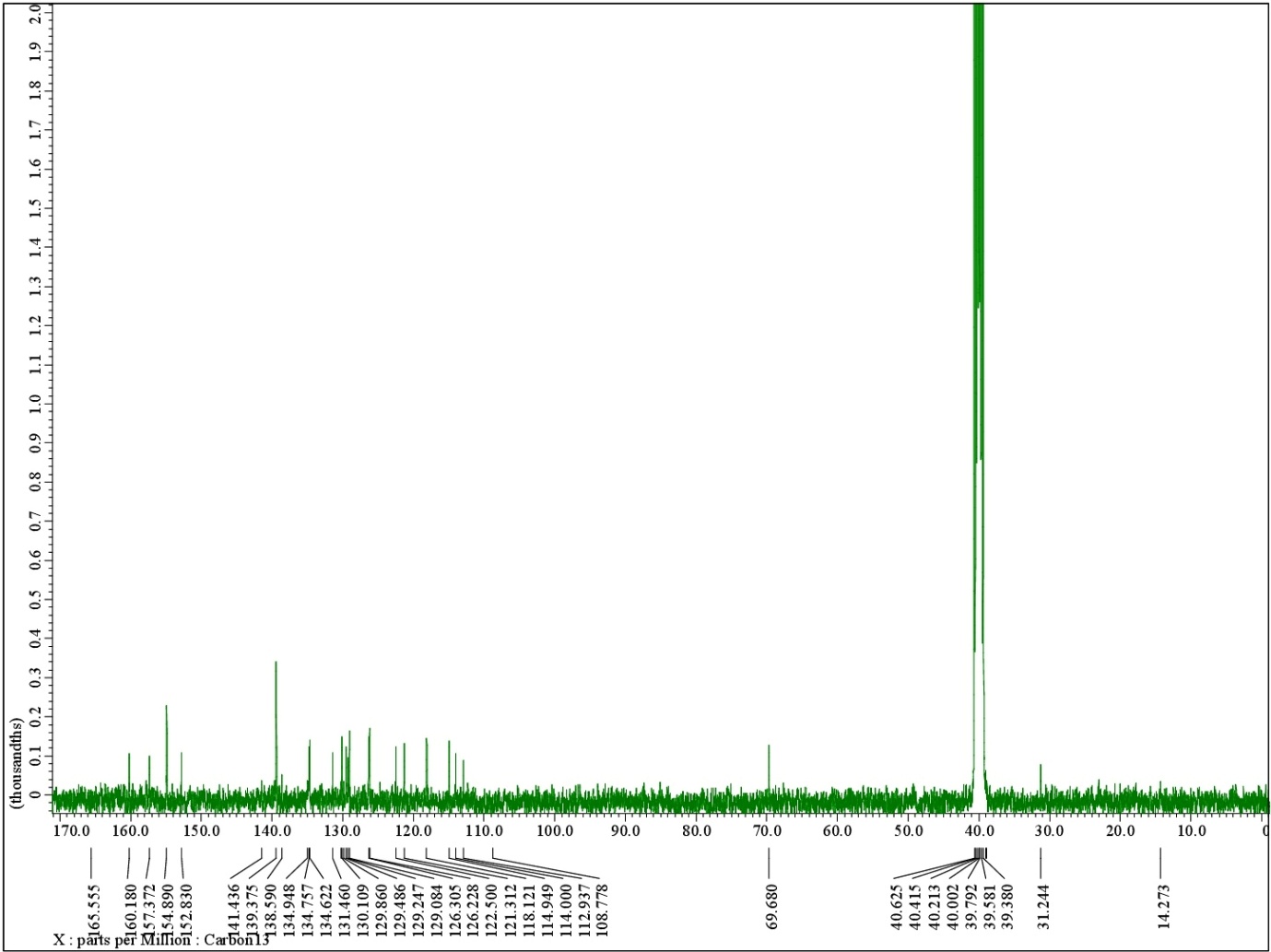


**Spectrum 18:** ^13^C NMR Spectrum of compound **(3e)** in DMSO-d_6_


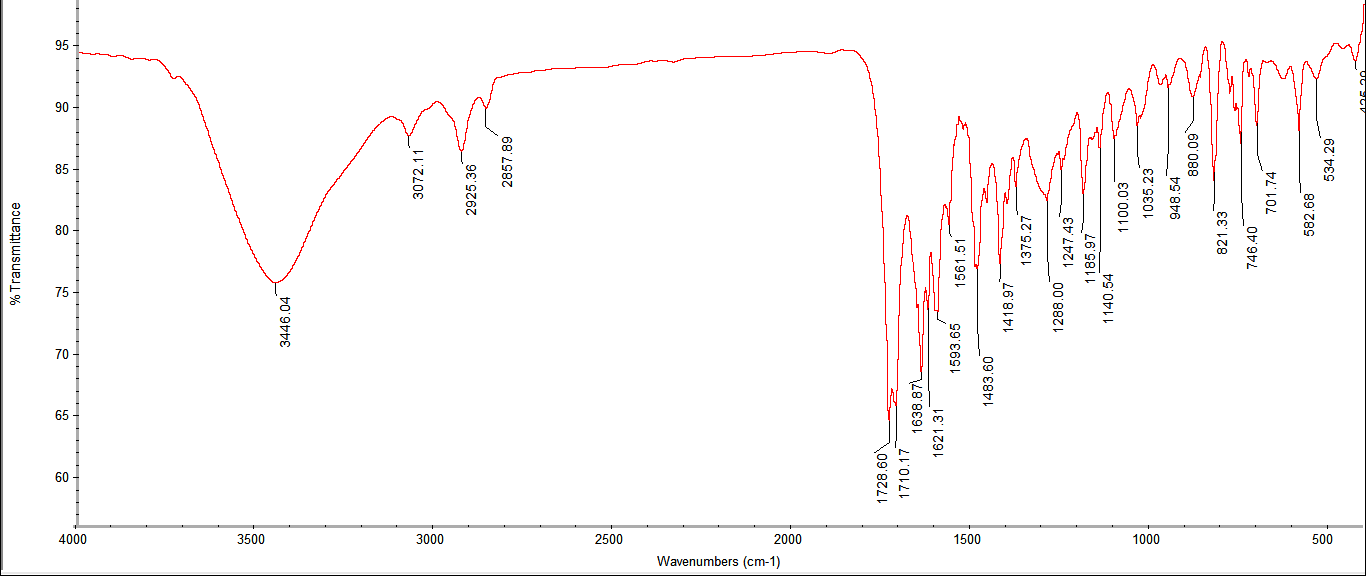


**Spectrum 19:** IR Spectrum of compound **(3e)**

**
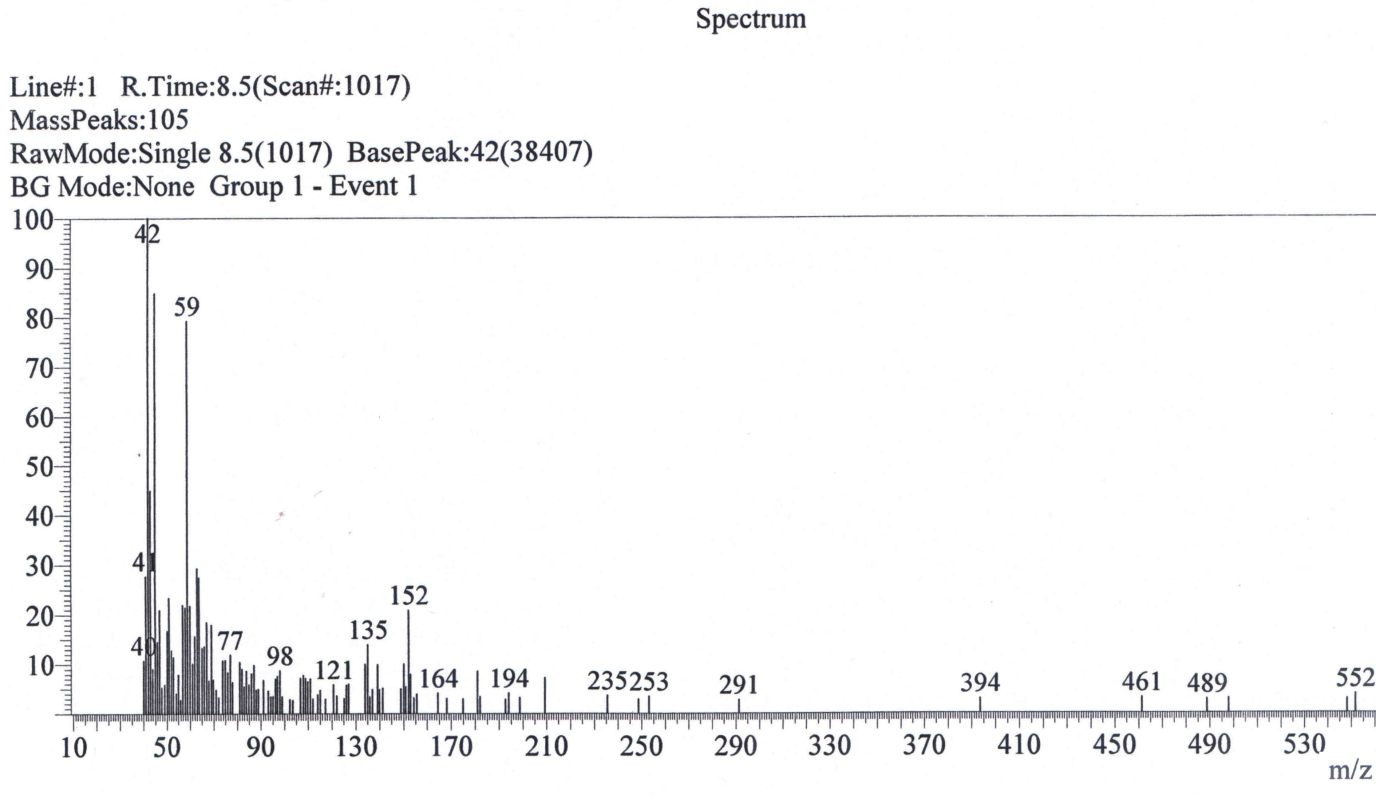
**

**Spectrum 20:** Mass Spectrum of compound **(3e)**


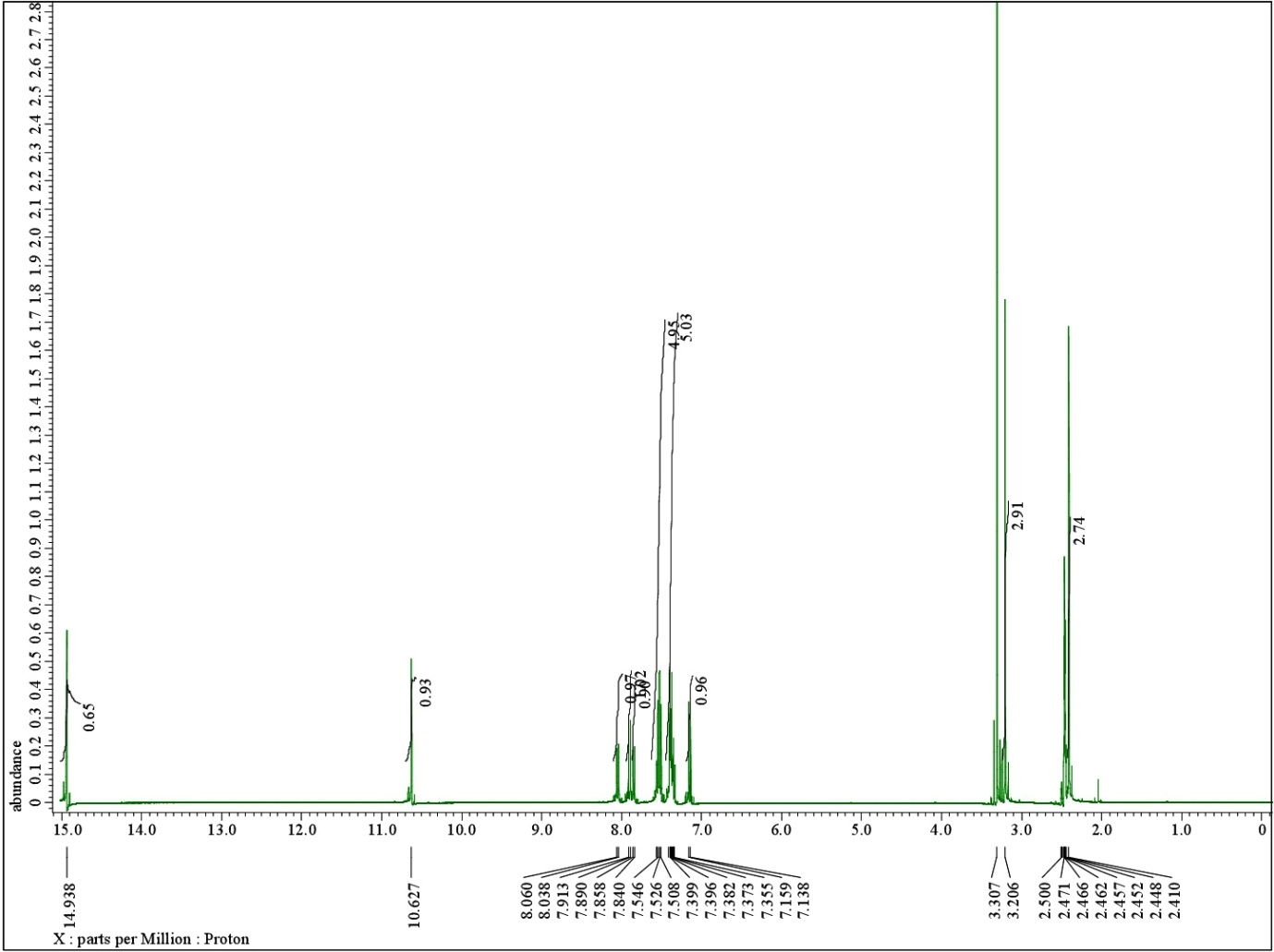


**Spectrum 21:** ^1^H NMR Spectrum of compound **(3f)** in DMSO-d_6_


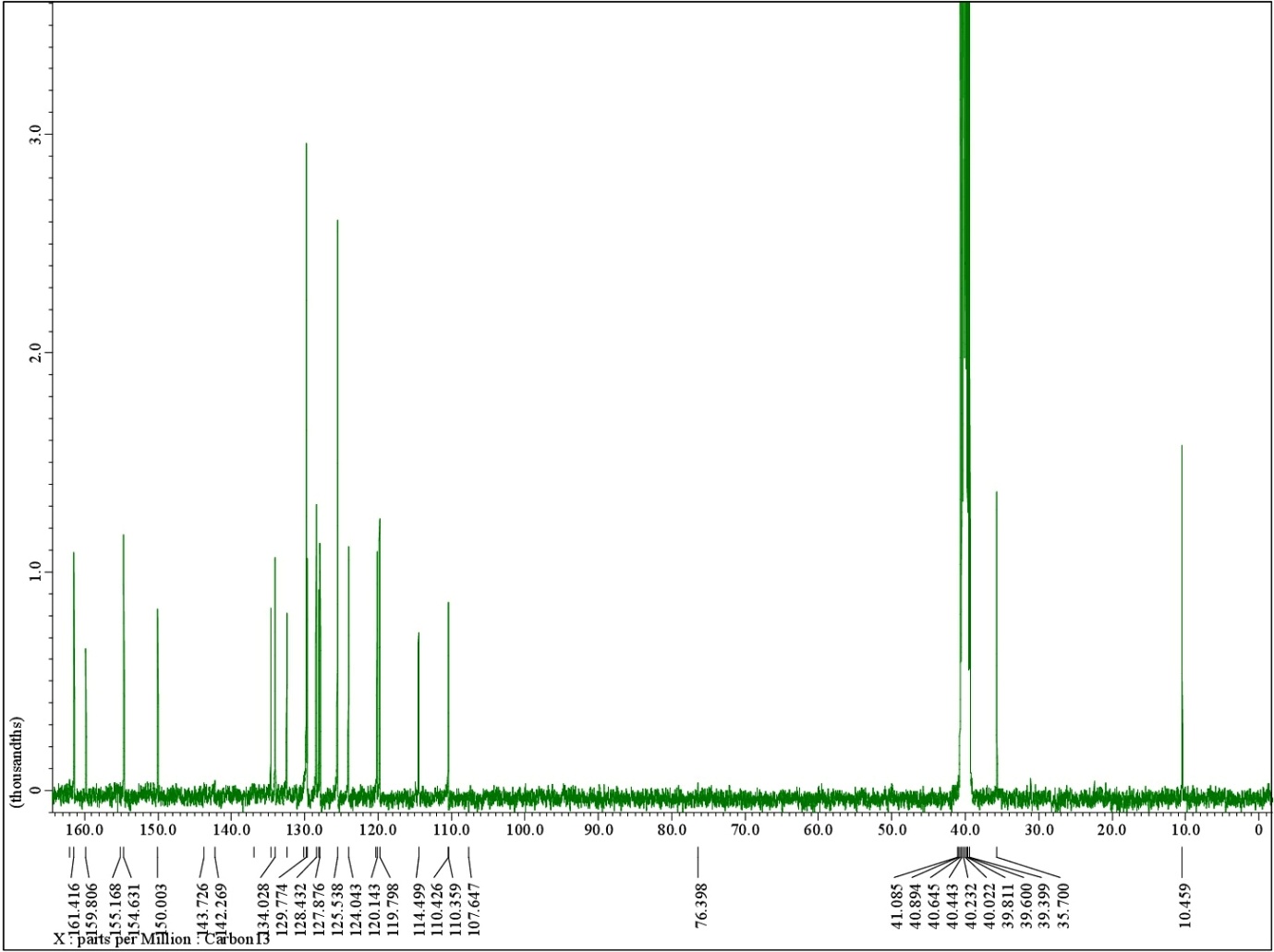


**Spectrum 22:** ^13^C NMR Spectrum of compound **(3f)** in DMSO-d_6_


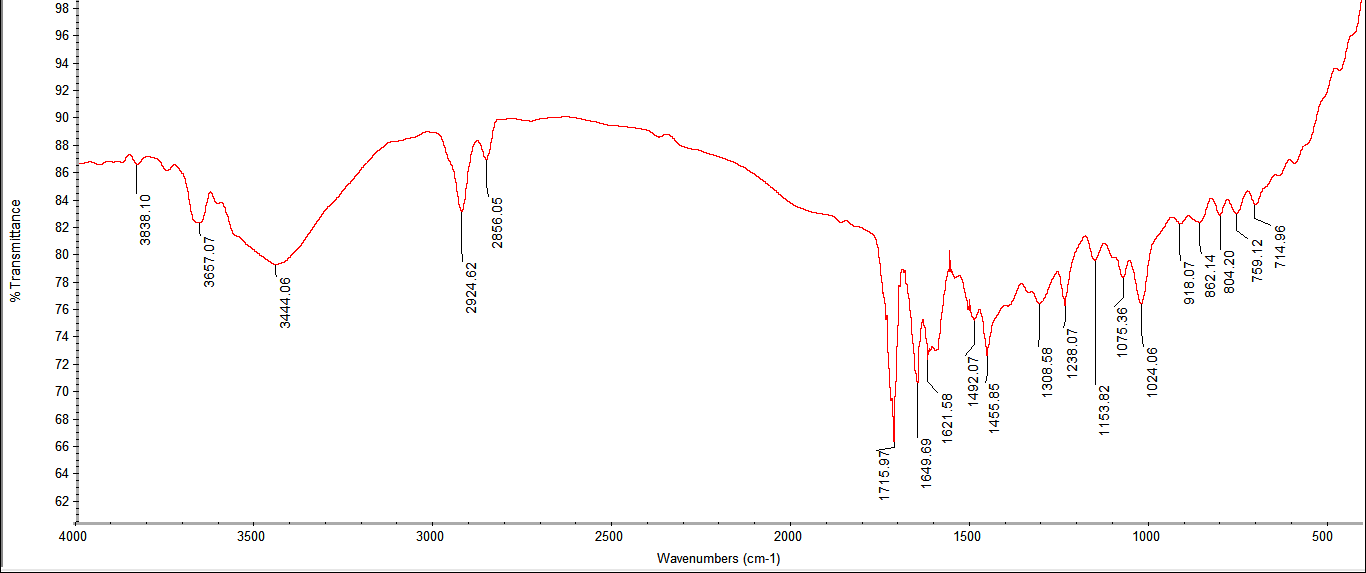


**Spectrum 23:** IR Spectrum of compound **(3f)**

**
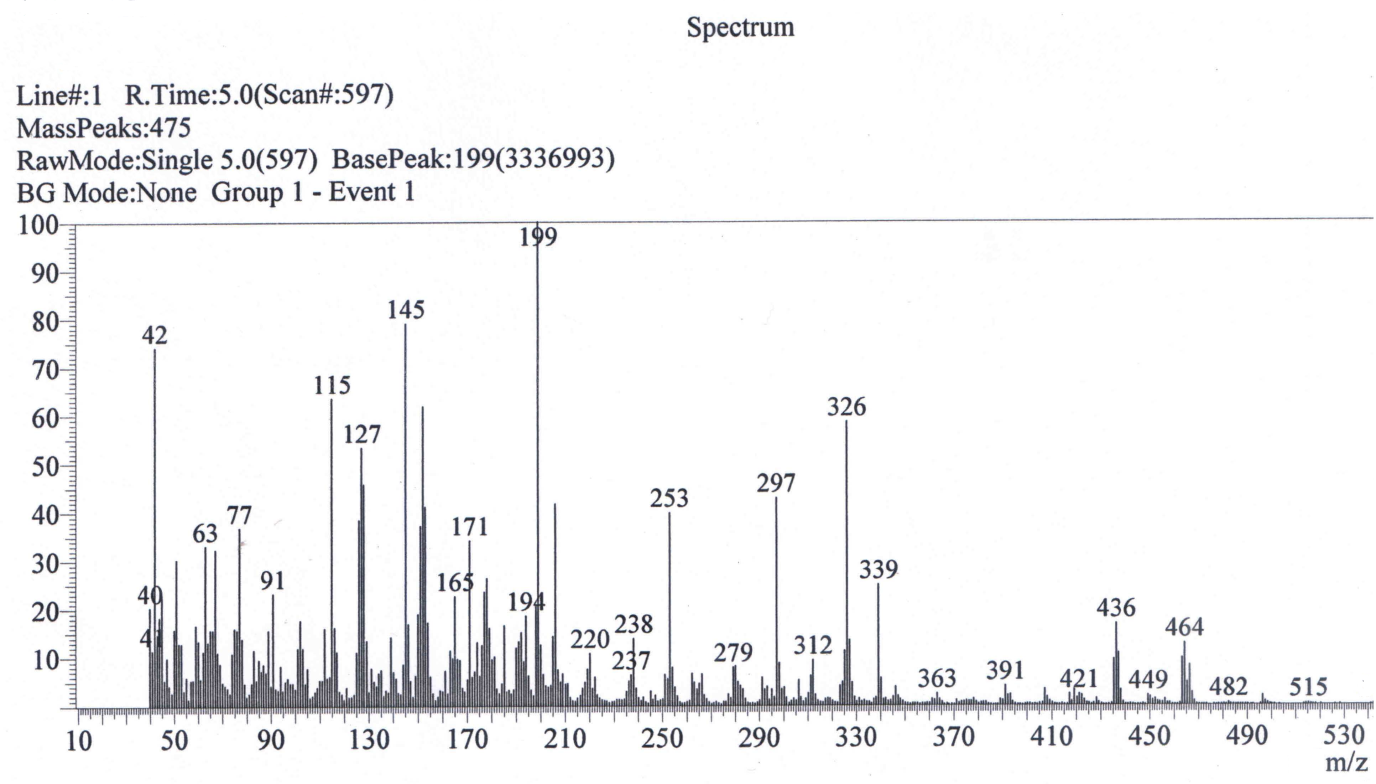
**

**Spectrum 24:** Mass Spectrum of compound **(3f)**


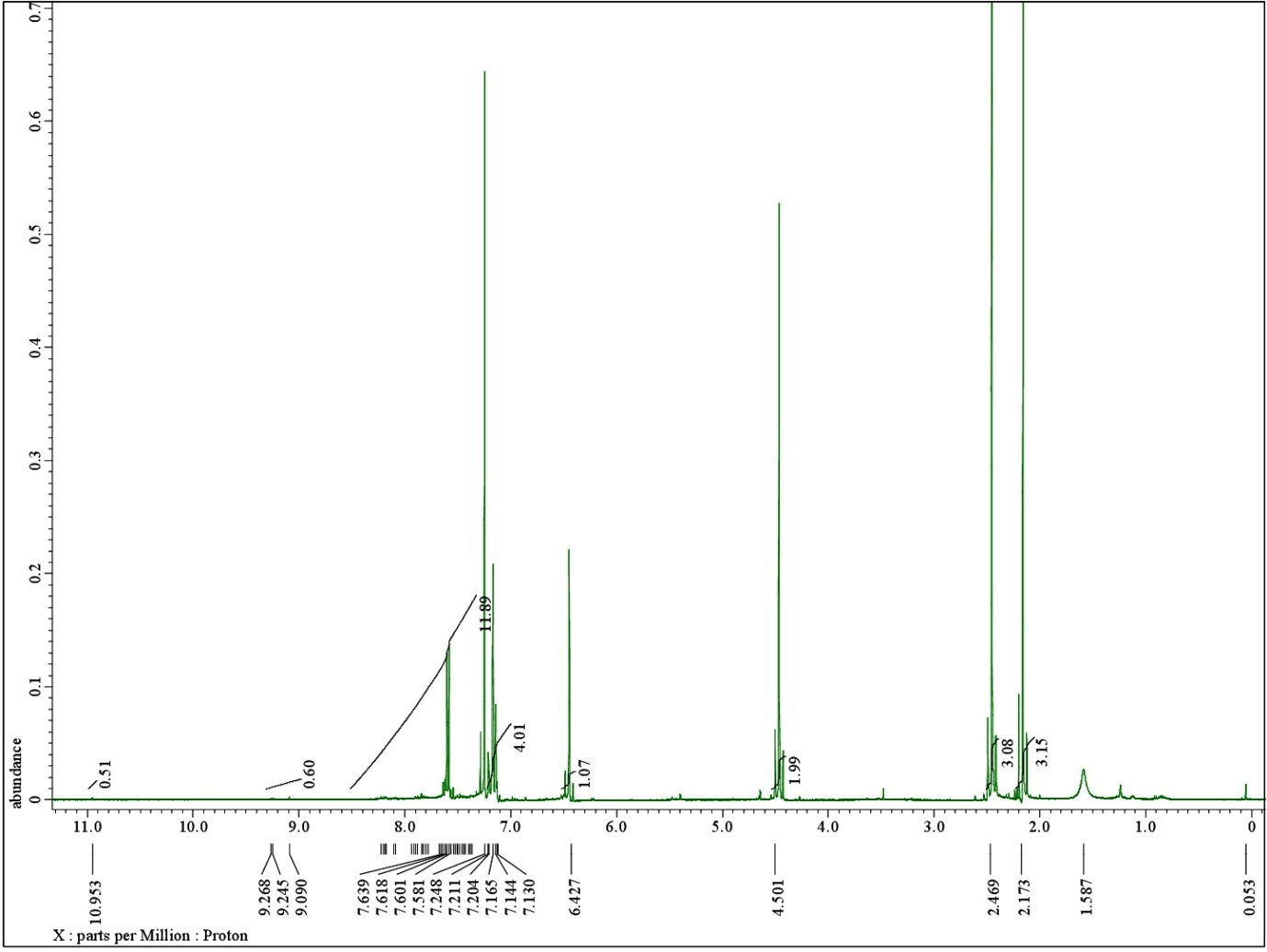


**Spectrum 25:** ^1^H NMR Spectrum of compound **(3g)** in DMSO-d_6_


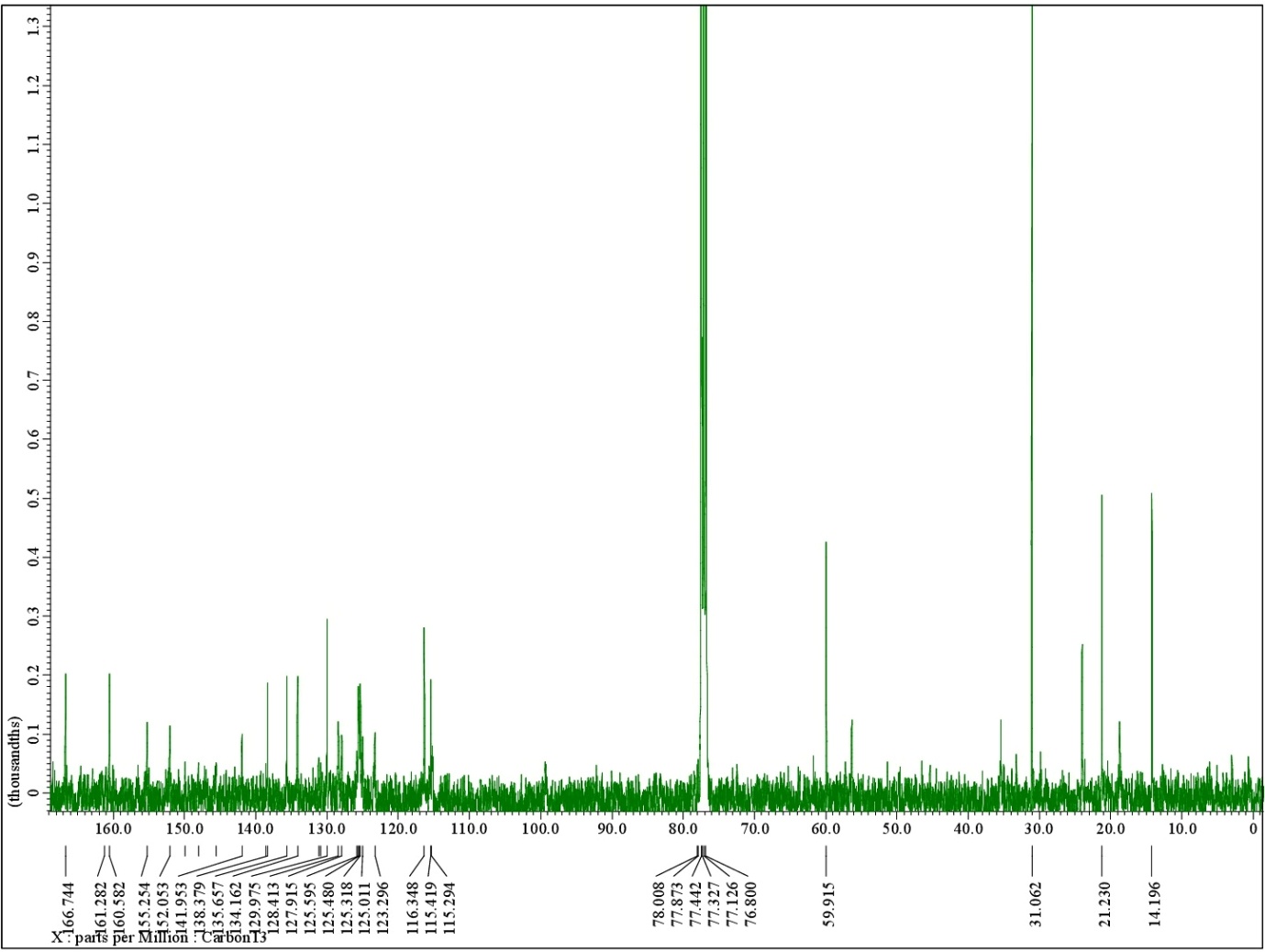


**Spectrum 26:** ^13^C NMR Spectrum of compound **(3g)** in DMSO-d_6_


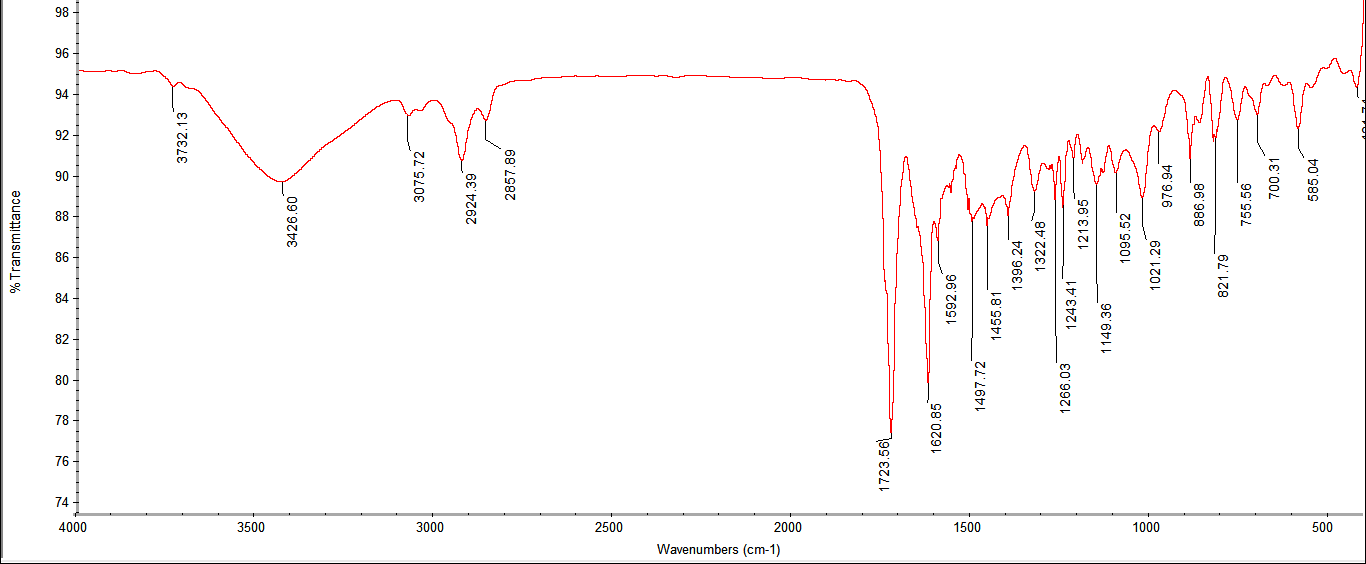


**Spectrum 27:** IR Spectrum of compound **(3g)**

**
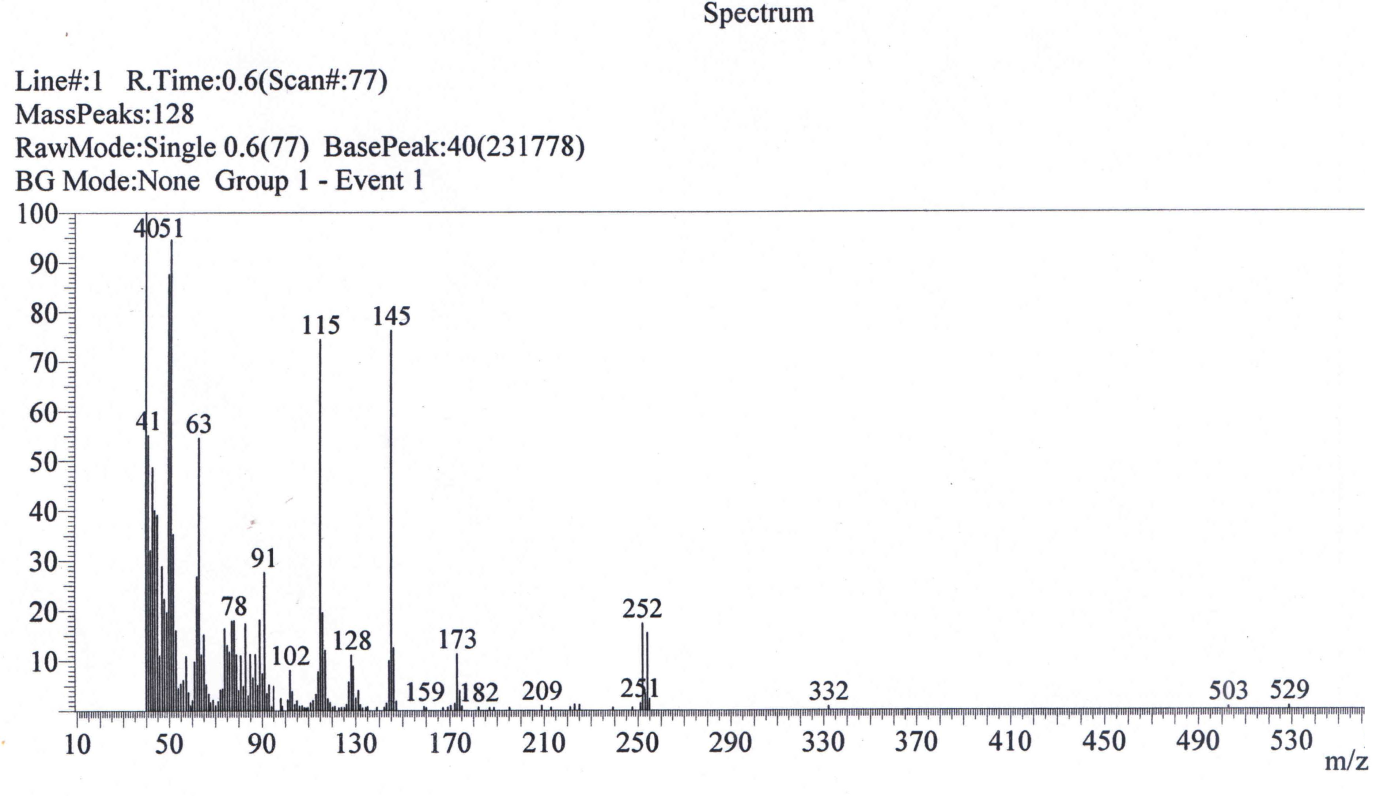
**

**Spectrum 28:** Mass Spectrum of compound **(3g)**


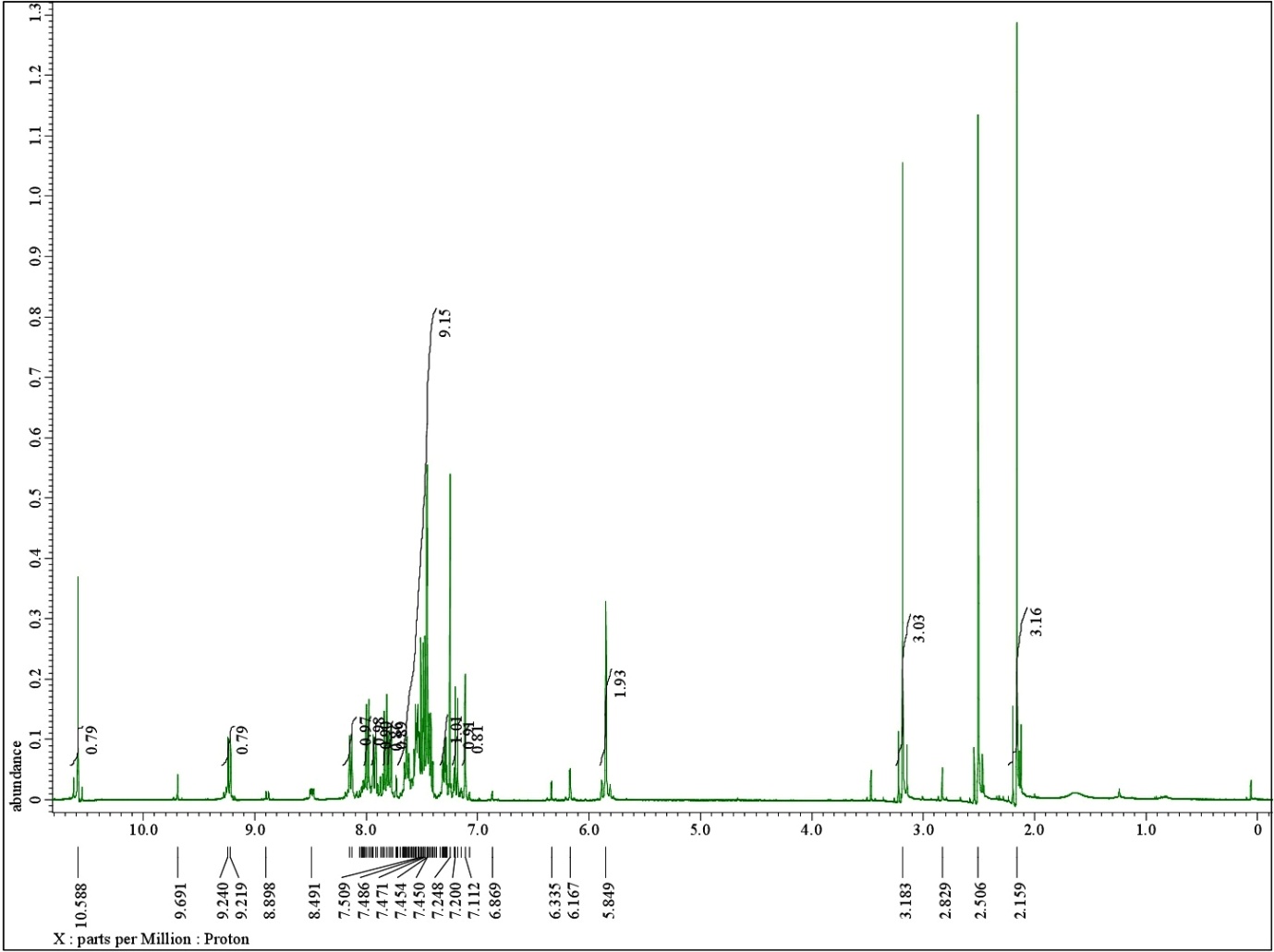


**Spectrum 29:** ^1^H NMR Spectrum of compound **(3h)** in DMSO-d_6_


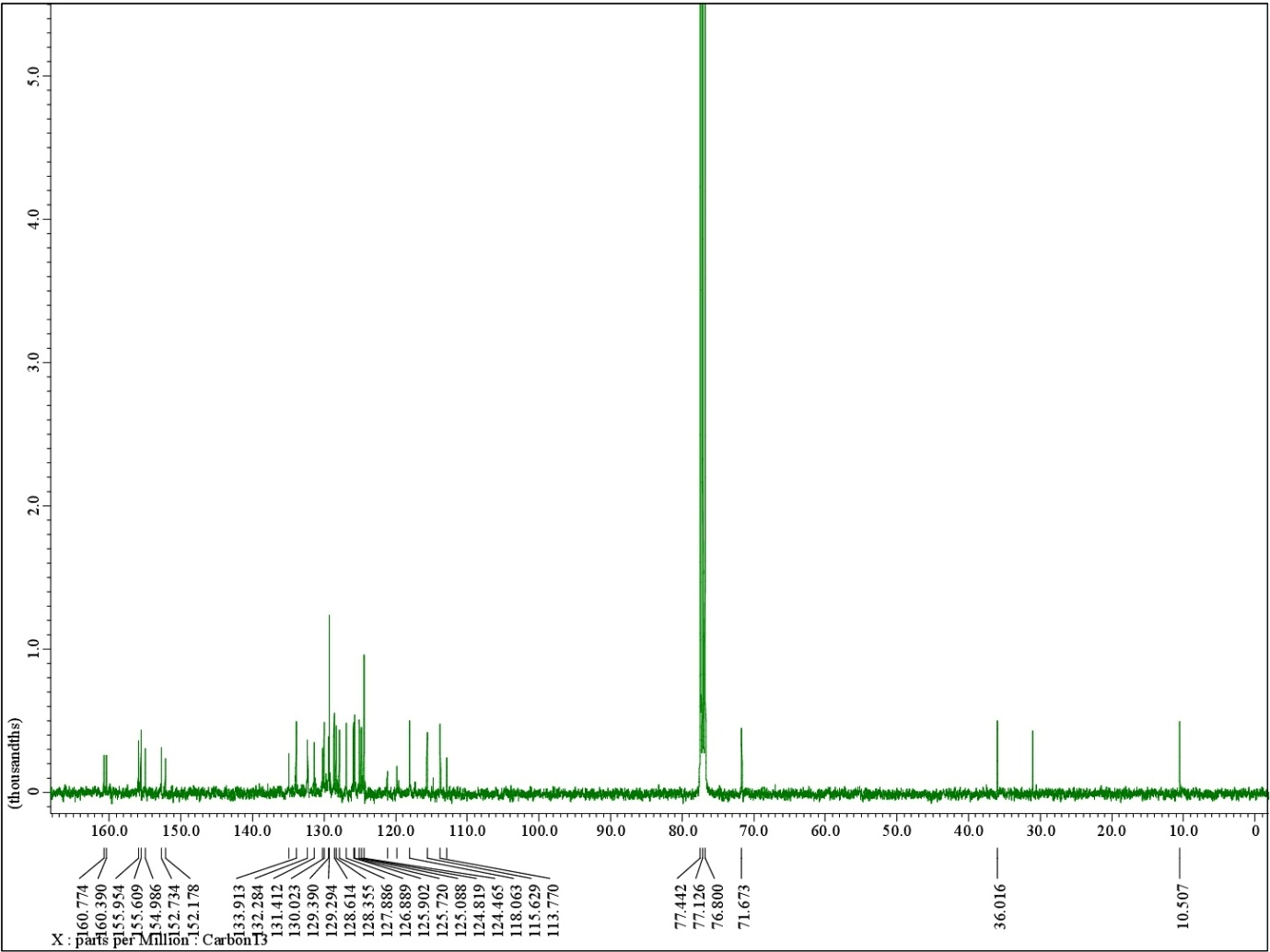


**Spectrum 30:** ^13^C NMR Spectrum of compound **(3h)** in DMSO-d_6_


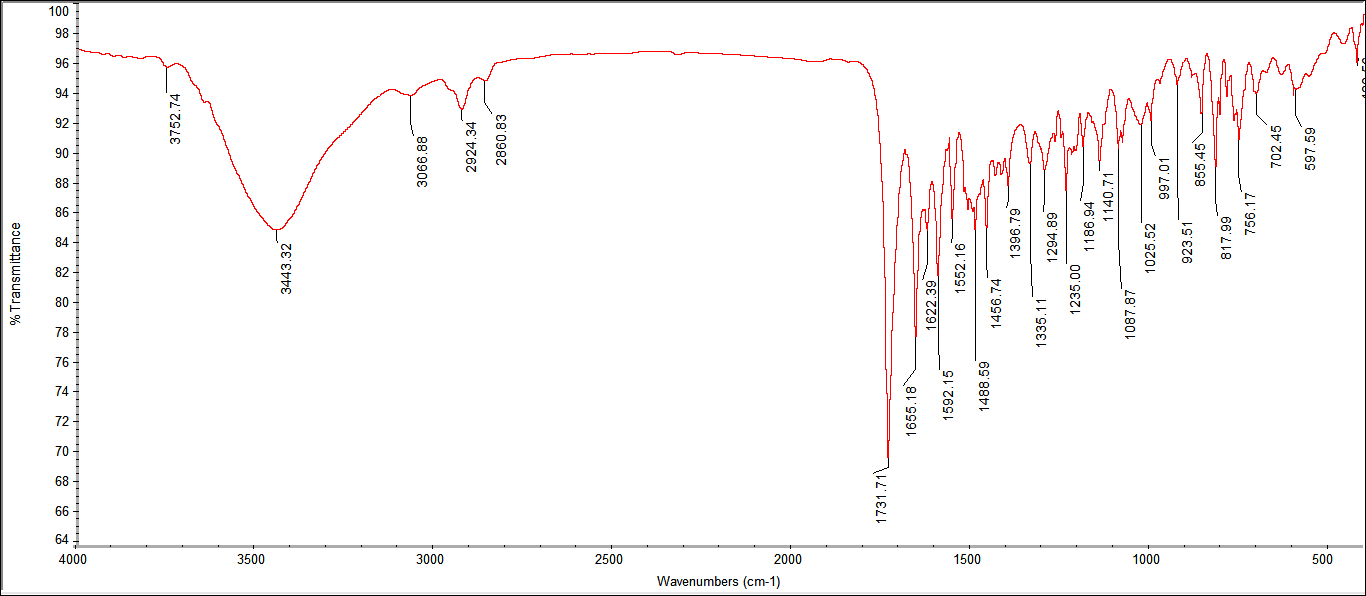


**Spectrum 31:** IR Spectrum of compound **(3h)**

**
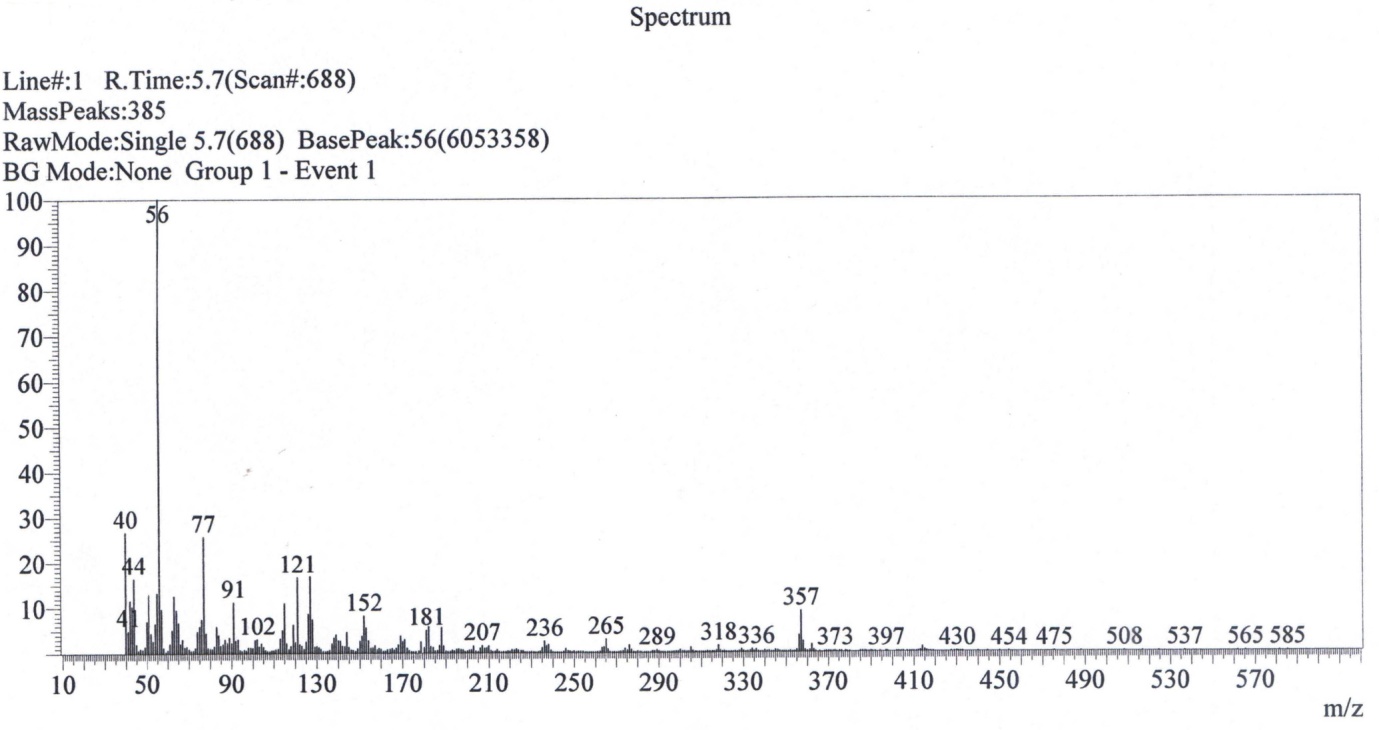
**

**Spectrum 32:** Mass Spectrum of compound **(3h)**


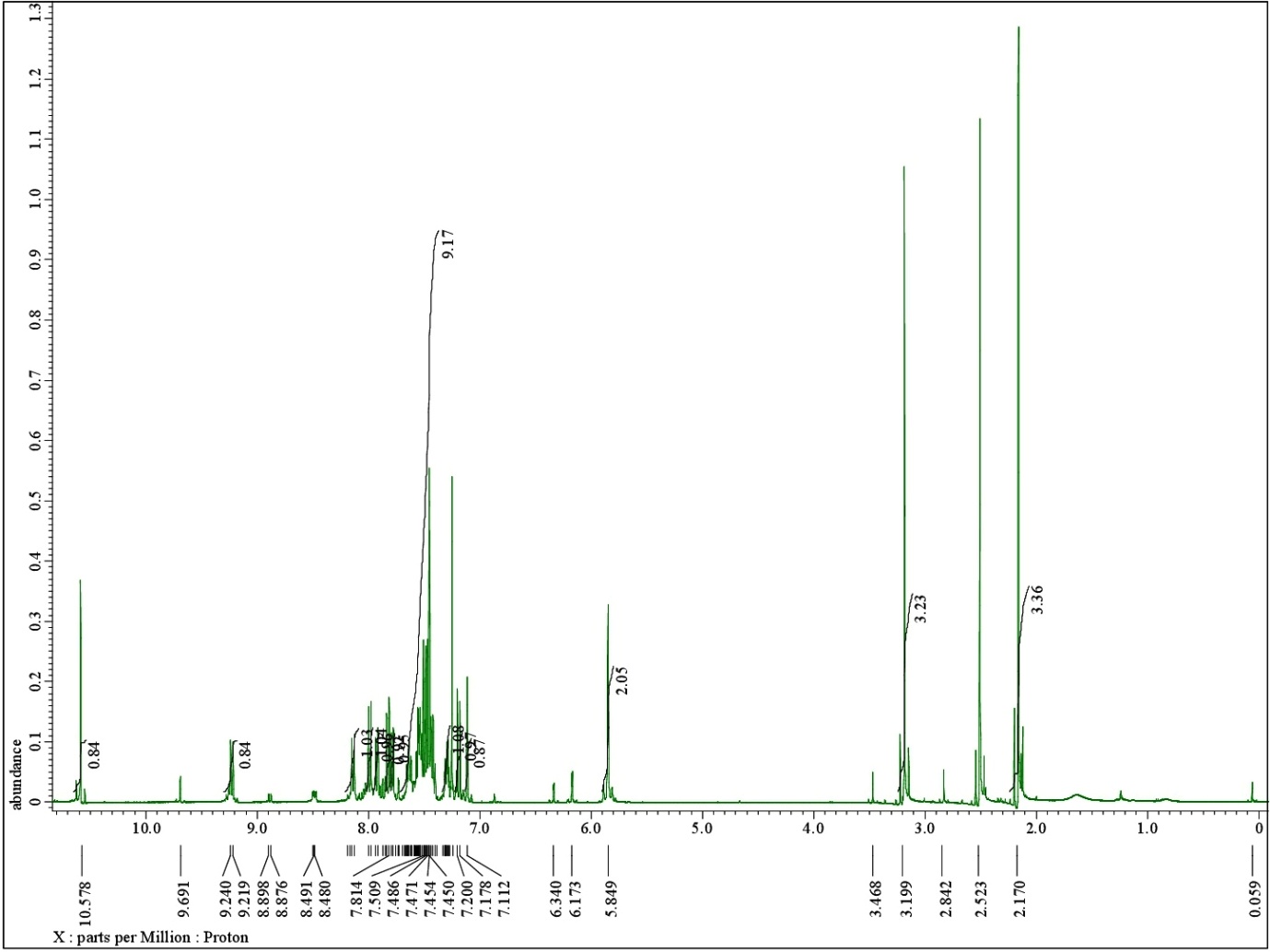


**Spectrum 33:** ^1^H NMR Spectrum of compound **(3i)** in CDCl_3_


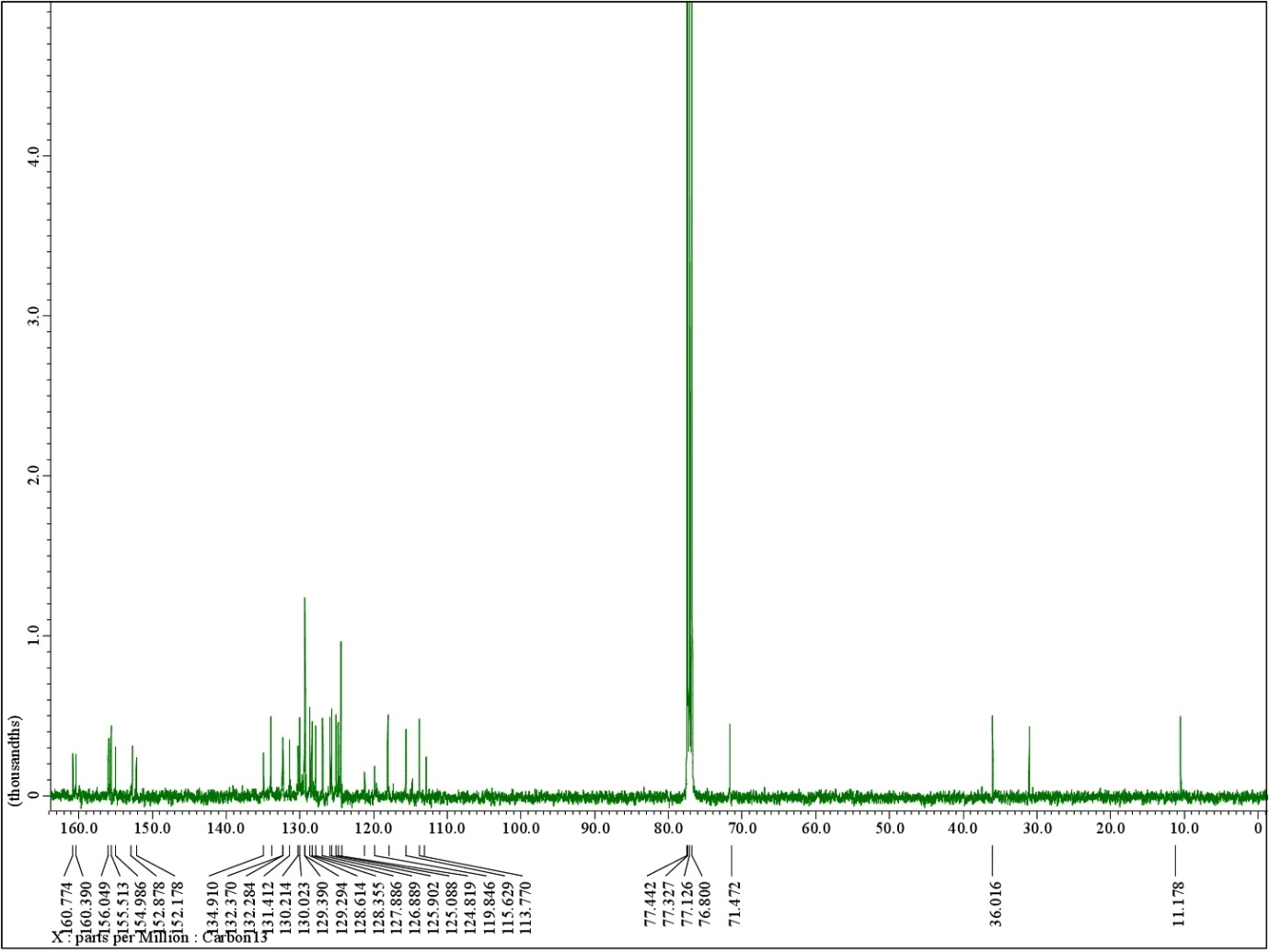


**Spectrum 34:** ^13^C NMR Spectrum of compound **(3i)** in CDCl_3_


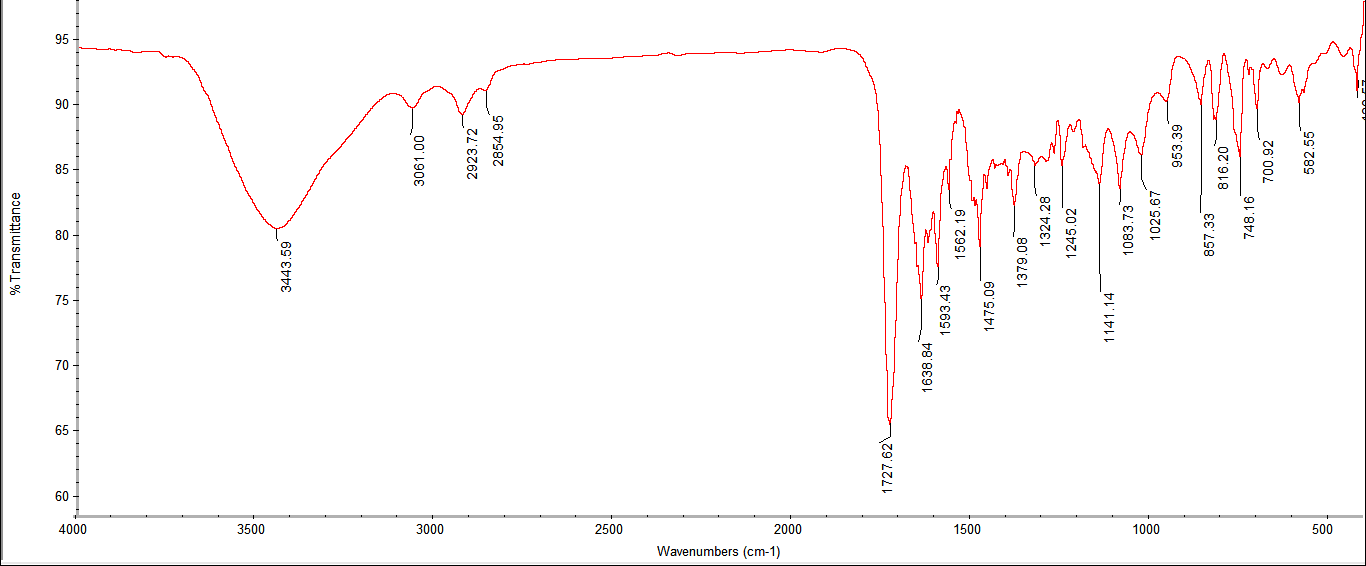


**Spectrum 35:** IR Spectrum of compound **(3i)**

**
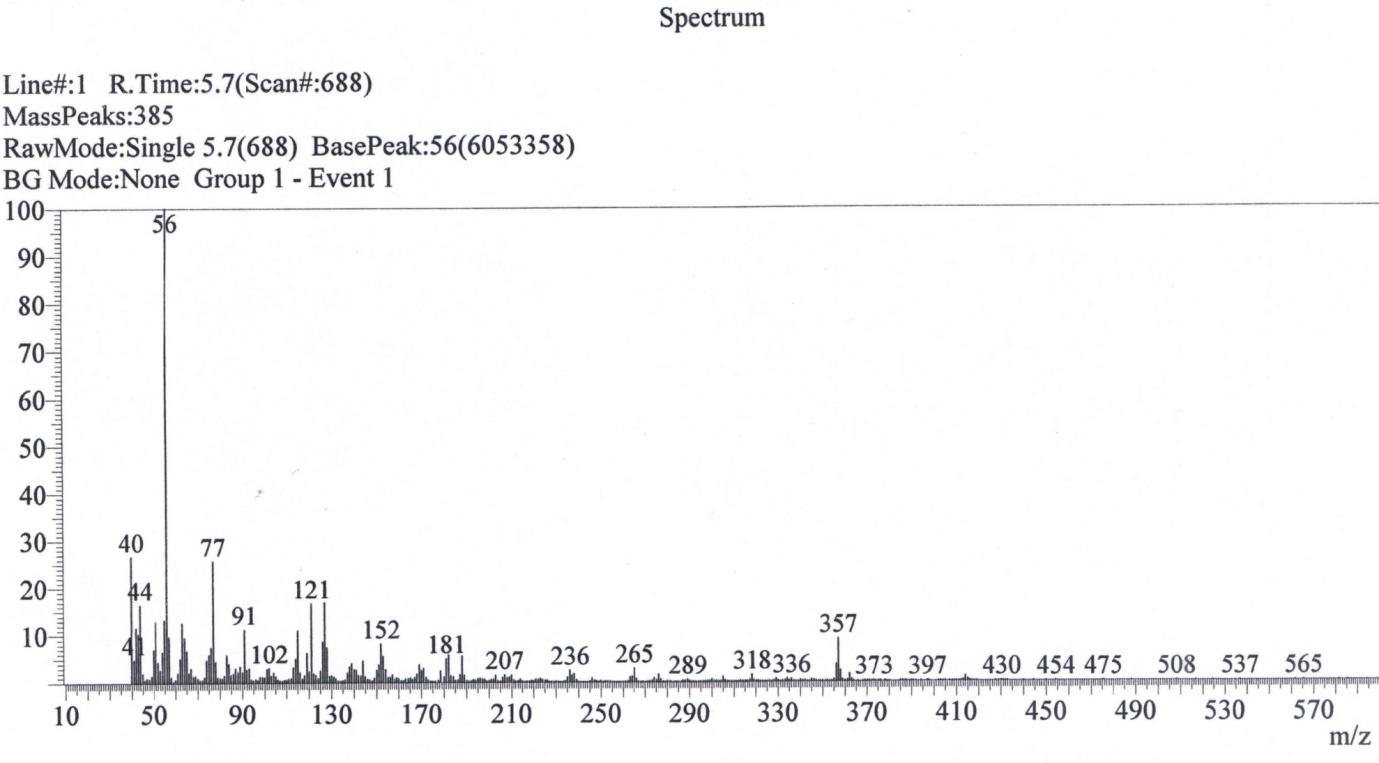
**

**Spectrum 36:** Mass Spectrum of compound **(3i)**
